# Metagenomic Insights into the Fibrolytic Microbiome in Yak Rumen

**DOI:** 10.1371/journal.pone.0040430

**Published:** 2012-07-13

**Authors:** Xin Dai, Yaxin Zhu, Yingfeng Luo, Lei Song, Di Liu, Li Liu, Furong Chen, Min Wang, Jiabao Li, Xiaowei Zeng, Zhiyang Dong, Songnian Hu, Lingyan Li, Jian Xu, Li Huang, Xiuzhu Dong

**Affiliations:** 1 State Key Laboratory of Microbial Resources, Institute of Microbiology, Chinese Academy of Sciences, Beijing, China; 2 CAS Key Laboratory of Genome Sciences and Information, Beijing Institute of Genomics, Chinese Academy of Sciences, Beijing, China; 3 Bioinformation Center, Institute of Microbiology, Chinese Academy of Sciences, Beijing, China; 4 Qingdao Institute of Bioenergy and Bioprocess Technology, Chinese Academy of Sciences, Qingdao, China; Missouri University of Science and Technology, United States of America

## Abstract

The rumen hosts one of the most efficient microbial systems for degrading plant cell walls, yet the predominant cellulolytic proteins and fibrolytic mechanism(s) remain elusive. Here we investigated the cellulolytic microbiome of the yak rumen by using a combination of metagenome-based and bacterial artificial chromosome (BAC)-based functional screening approaches. Totally 223 fibrolytic BAC clones were pyrosequenced and 10,070 ORFs were identified. Among them 150 were annotated as the glycoside hydrolase (GH) genes for fibrolytic proteins, and the majority (69%) of them were clustered or linked with genes encoding related functions. Among the 35 fibrolytic contigs of >10 Kb in length, 25 were derived from Bacteroidetes and four from Firmicutes. Coverage analysis indicated that the fibrolytic genes on most Bacteroidetes-contigs were abundantly represented in the metagenomic sequences, and they were frequently linked with genes encoding SusC/SusD-type outer-membrane proteins. GH5, GH9, and GH10 cellulase/hemicellulase genes were predominant, but no GH48 exocellulase gene was found. Most (85%) of the cellulase and hemicellulase proteins possessed a signal peptide; only a few carried carbohydrate-binding modules, and no cellulosomal domains were detected. These findings suggest that the SucC/SucD-involving mechanism, instead of one based on cellulosomes or the free-enzyme system, serves a major role in lignocellulose degradation in yak rumen. Genes encoding an endoglucanase of a novel GH5 subfamily occurred frequently in the metagenome, and the recombinant proteins encoded by the genes displayed moderate Avicelase in addition to endoglucanase activities, suggesting their important contribution to lignocellulose degradation in the exocellulase-scarce rumen.

## Introduction

The rumen is a unique natural habitat that has evolved into a complex and efficient system for lignocellulose degradation. Over the past few decades, considerable efforts have been made to isolate fibrolytic bacteria and identify lignocellulose-degrading enzymes from the rumen of a variety of herbivores. *Fibrobacter succinogenes*, *Ruminococcus albus*, *Ruminococcus flavefaciens, Butyrivibrio fibrisolvens*, and *Prevotella ruminicola* are believed to be the predominant lignocellulose degraders in the rumen [1]. A large number of cellulases, hemicellulases, and esterases have been purified from these organisms. Complexity of the lignocellulose-degrading enzymes in these bacteria has been confirmed by genome sequencing [2], [3], [4]. More recently, metagenomic studies demonstrated that in the rumen, plant cell wall-degrading enzymes exist in far greater diversity than previously believed [5], [6]. Enzymatic and protein-structure studies indicated that free-enzyme system and cellulosomes are the two main lignocellulose-degrading mechanisms used by cultured fibrolytic bacteria [7]. However, the primary cellulolytic systems operating *in situ* in the rumen remain elusive.

The yak (*Bos grunniens*), a large ruminant of the bovine family with an adult body weight of over 1,000 kg, primarily inhabits the Qinghai–Tibetan Plateau, China. The animal grazes exclusively on grasses, straw, and lichens. Surveys of the 16S rRNA gene diversity showed that microorganisms in the yak rumen were less diverse than those in cattle rumen; however a greater proportion was uncultured in the former than in the latter [8]. Thus the yak rumen may harbor a unique microbiome for efficient conversion of fibrous materials.

Sequenced fibrolytic rumen and gut bacterial genomes revealed that the cellulases/hemicellulases and functionally related genes were frequently physically clustered or linked on the genome [2], [9]. In the present report, we investigate fibrolytic genes and gene clusters in the rumen microbiome of yak fed on wheat stalk by constructing a large-insert bacterial artificial chromosome (BAC) library for the rumen metagenomic DNA and subsequent pyrosequencing of the inserts of clones active in a fibrolytic enzyme screening. In parallel, we performed pyrosequencing and Solexa sequencing on the metagenomic DNA sample shared with the BAC library to estimate the occurrence frequencies of the BAC-retrieved fibrolytic genes in the entire microbiome of the yak rumen. With the method developed in this work, we were able not only to obtain the cellulolytic genes in their entire length for domain analysis but also to assemble large contigs, permitting insight into the organizational patterns of fibrolytic gene clusters that are indicative of the metabolic and regulatory features of lignocellulose degradation mechanisms in yak rumen.

## Results

### Fibrolytic Genes and Gene Clusters Retrieved through Construction of a BAC Clone Library for Yak Rumen Microbiome

**Figure 1:**
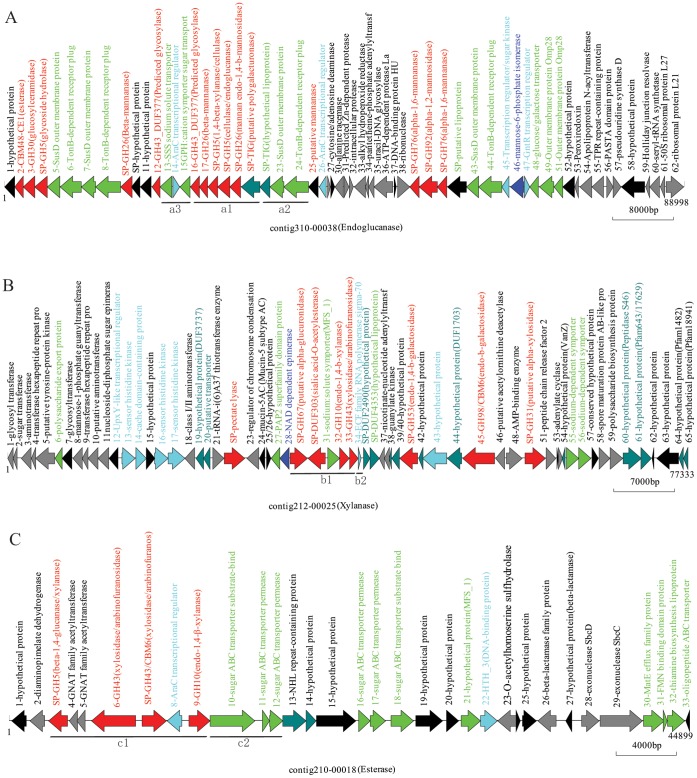
Organization of genes on representative contigs retrieved from the fibrolytic BAC clones. Red, fibrolytic enzymes; Blue, regulatory proteins; Light green, membrane protein/transporter system; Grey, diverse functions; Dark green, hypothetical proteins with the domains deposited in Pfam database; Black, hypothetical protein without known domains. SP, signal peptide. (A) Contig 310-00038: a Bacteroidetes-derived sequence from a BAC clone with carboxylmethyl cellulase activity. (B) Contig 212-00025: a Bacteroidetes-derived sequence from a BAC clone with xylanase activity. (C) Contig 210-00018: a Firmicutes-derived sequence from a BAC clone with lipase activity. Lower case letters refer to the gene fragments discussed in the text. Scale indicates the nucleotide base pairs.

To obtain the cellulolytic genes in their entire length for architectural characterization of the proteins and the organizational patterns of fibrolytic gene clusters, we constructed a large insert BAC clone library of yak rumen metagenome. The library consists of 76,000 BAC clones with an average insert size of ∼55 kb and, therefore, contains ∼4.2 Gbp of cloned DNA. A total of 9,600 randomly selected BAC clones were screened for cellulase (Figure S1A), xylanase (Figure S1B), and carbohydrate esterase activities (Figure S1C), yielding a total of 223 positive clones. The inserts in all of the 223 BAC clones were pyrosequenced and assembled. In all, 838,584 reads, or 299.9 Mbp of DNA sequence were generated, representing a 21-fold theoretical coverage of the total DNA inserts. Sequence assembly yielded 4,936 contigs with 10,070 ORFs (Table S1). Among these ORFs, 150 non-redundant full-length genes were predicted to encode a fibrolytic enzyme (E-value <1e^−5^, Table S2), and the majority of them (68.7%) were clustered or linked with genes encoding related functions, and situated on 35 fibrolytic contigs with length >10 kb (Figure 1 and Figure S2).

### Genomic Organizations of the Fibrolytic Genes

To reveal the metabolic and regulatory features of lignocellulose degradation mechanisms in yak rumen, we analyzed the fibrolytic genes organization on the 35 fibrolytic contigs. The majority of the GH protein genes situated in the large contigs were from Bacteroidetes (25 of 35), and the rest were from Firmicutes (4 of 35), Fibrobacteres (2 of 35), and other phyla (4 of 35). The lignocellulase gene contigs from Bacteroidetes, as shown in Figure 1A, displayed one of the following characteristics. First, a variety of GH genes relevant to plant cell wall degradation formed a cluster, e. g., GH43-GH26-GH5-GH5-GH26 and a putative polygalacturonase gene (Fragment a1 of Figure 1A), suggesting that they were co-transcripted and functioned synergistically. Second, *susD* (starch utilization system), which encodes an outer-membrane protein, and the putative *susC*-like, which encodes an integral-membrane protein (Fragment a2 of Figure 1A), followed by a gene encoding an unknown lipoprotein, were located directly upstream of a cellulolytic gene cluster, implying that the SusD may be involved in cellulolysis. Third, many of the SusC/SusD-like encoding genes were linked with a major facilitator superfamily (MFS) gene (Fragment a3 of Figure 1A) or cation sugar symporter, and fewer with ATP binding cassette (ABC) transporters, suggesting that the MFS, probably together with SusC, plays a role in importing the oligosaccharides produced during fibrolysis. Forth, most of the putative cellulose genes were clustered with the transcription regulator AraC or LytR (Fragment a3 of Figure 1A), whereas only some of the putative hemicellulase or esterase genes (Fragment b1 of Figure 1B) were linked to an extra-cellular function (ECF) sigma factor (Fragment b2 of Figure 1B and Figure S2A).

The genomic organization of fibrolytic genes on contigs derived from Firmicutes (Figure 1C and Figure S2B) was somewhat different. Although clustering of GH genes with an AraC transcriptional regulator was also found (e.g., GH5-GH43-GH43-AraC-GH10, fragment c1 of Figure 1C), ABC transporters or PTSII genes, instead of the SusC/SusD-like genes (Fragment c2 of Figure 1C), were located adjacent to GH genes in some cases, suggesting that different types of sugar transporters, and thus different preferred oligosaccharide substrates, were used by the Firmicutes and Bacteroidetes in the yak rumen.

### Abundance of the Fibrolytic Contigs in the Yak Rumen Microbiome

**Figure 2:**
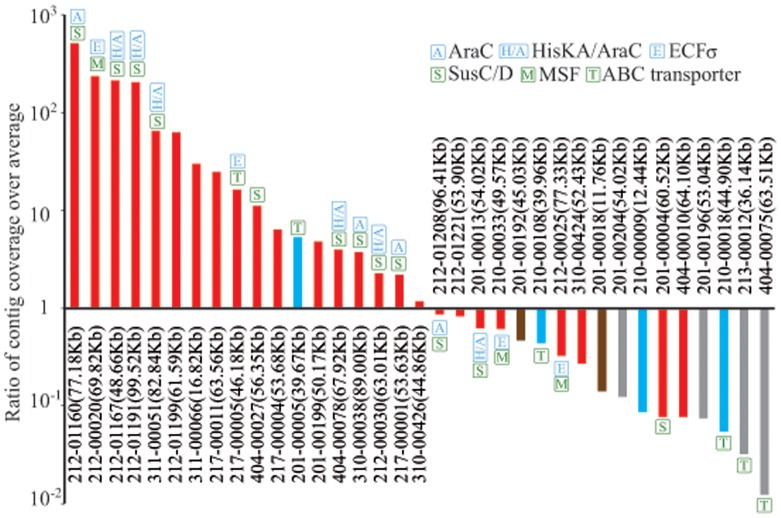
Sequence coverage of the 35 BAC-assembled contigs over the average coverage of total genes. In the parentheses and on the top of each bar indicate the contig length and the fibrolytic related genes, respectively. Red, Bacteriodetes; Blue, Firmicutes; Brown, Fibrobacter; Grey, Unclassified.

To assess the abundance of fibrolytic genes, all of the metagenomic Solexa reads were aligned to BAC-derived fibrolytic contigs as well as other metagenomic contigs (see Materials and Methods). An average sequence coverage for the metagenomic contigs was 4.0, while that for the 35 BAC-derived contigs reached 7.1, indicating that the fibrolytic genes were highly abundant in yak rumen. Among the 35 contigs, the sequence coverage of 18 contigs was above the average for the metagenomic contigs, whereas the coverage of 8 contigs was 10 to 100-fold lower than the average (Figure 2). It appears that the characteristics of the 35 BAC-derived fibrolytic contigs are representative of those of the fibrolytic microbiome in the yak rumen. Our results suggest that a combination of functional screening of a BAC library and pyrosequencing provides an effective approach for retrieving functional genes and gene clusters.

### Diversity of Fibrolytic Enzymes in Yak Rumen

To determine the diversity of plant cell wall-degrading enzymes in the yak rumen microbiome, we performed protein domain analysis on the ORFs present in the metagenome using Pfam database [10], and showed the proteins belonging to GH families in Table S3. According to the identified enzymatic activities of the proteins from various GH families (Table S4), we showed the genes for the plant-cell-wall-targeting GH proteins that retrieved from yak rumen in this work and other animal guts in Table 1. the GH profile in yak rumen was similar to those reported for other animal gut microbiomes [5], [6], [11], [12], except that GH53 (exclusively galactanases), GH67 (including α-glucuronidases) and GH43 (including various oligosaccharides-degrading enzymes) seemed to exist in abundance only in the yak rumen. Similar to the bovine rumen, GH9 proteins and probably GH5 were the most abundant endoglucanases, and GH10 and GH26 as xylanases and mannanase were the predominant hemicellulases in the yak rumen (Table 1). GH11 hemicellulases were prevalent in the termite gut; however, they occurred at a lower frequency in the yak rumen. The prevalent oligosaccharide-degrading enzymes fall into three families, i.e., GH43, GH3 and GH2. As has been reported for the rumen or gut microbiomes in other animals [5], [6], [12], genes encoding cellobiohydrolases or proteins of the GH48 family, key components involved in natural lignocellulose degradation by cultured cellulolytic microorganisms [13], [14], [15], were barely detected in the yak rumen microbiome (32 reads). This finding suggests the presence of other GH proteins with exocellulase activity in yak rumen.

**Table 1:** Profiles of GH proteins targeting plant cell wall in rumen and gut microbiomes^1^.

	Yak rumen^2^	Cow rumen [5]	Bovine rumen [6]	Macropod Gut [11]	Termite Gut [12]
**Cellulases**					
GH5	1302(12)	1451	7	10 (14)	56
GH6	0(0)	0	0	0 (1)	0
GH7	0(0)	1	0	0 (0)	0
GH9	767(7)	795	6	0 (2)	9
GH44	0(0)	–	0	0 (0)	6
GH45	13(1)	115	0	0 (0)	4
GH48	32(0)	3	0	0 (0)	0
Total	2114(20)	2365	13	10 (17)	75
**Endohemicellulases**					
GH8	174(6)	329	4	1 (1)	5
GH10	2664(14)	1025	7	11 (3)	46
GH11	244(0)	165	1	0 (0)	14
GH12	0(0)	0	0	0 (0)	0
GH26	537(9)	369	5	5 (8)	15
GH28	244(4)	472	5	2 (1)	6
GH53	1066(5)	–	17	9 (0)	12
Total	4929(38)	2360	39	28 (13)	98
**Debranching enzymes**					
GH51	0(9)	–	64	12 (1)	18
GH54	111(1)	–	1	0 (0)	0
GH62	0(0)	1	0	0 (0)	0
GH67	1090(2)	120	0	5 (0)	10
GH78	426(7)	1260	34	25 (0)	0
Total	1627(19)	1381	99	42 (1)	28
**Oligosaccharide-degrading enzymes**					
GH1	331(1)	253	10	61 (1)	22
GH2	942(13)	1436	186	24 (4)	23
GH3	5448(24)	2844	176	72 (3)	69
GH29	899(1)	939	74	2 (1)	0
GH35	468(0)	158	12	3 (1)	3
GH38	90(1)	272	17	3 (0)	11
GH39	159(1)	315	2	1 (0)	3
GH42	207(4)	374	11	8 (1)	24
GH43	2313(28)	–	61	10 (9)	16
GH52	0(0)	–	0	0 (0)	3
Total	10857(73)	6591	549	184 (20)	174
Total plant cell wall –targeting GHs	19527(150)	12697	700	264	375
Total GHs	37563(263)	27755	957	557	704

1 Numbers inside and outside parentheses are those retrieved from the constructed libraries and metagenomes from the same microbiomes, respectively.

2 Data inside the parentheses are the results of de-redundancy.

### Domain Architecture of the GH Proteins Retrieved from the Yak BAC Clones

Since all of the GH genes recovered from the sequenced BAC clones encoded full-length polypeptides, it is possible to predict their mode of action through protein domain analysis. Based on SignalP 3.0 prediction, we found that most of these fibrolytic proteins contained a signal peptide sequence, suggesting their extracellular site of action (Table S2). Pfam analysis indicates that 10% of the plant cell wall-targeting GH proteins carry a CBM; however, most of them are GH43 proteins, i.e., oligosaccharides-degrading enzymes.

CBM-appended fibrolytic proteins are known to be characteristic of the free-enzyme system employed by cellulolytic microbes [7]. CBM-encoding sequences were detected in both the metagenomic reads and the BAC inserts (Table S3); however, those believed to be the carbohydrate-binding domains appended at cellulases for binding the structured cellulose (i.e., CBM1, 2, and 3) appeared to exist infrequently. CBM6 and CBM47 were the exceptions; they occur in the metagenome and the BAC library at relatively high levels, and frequently occur at oligosaccharide-degrading and starch-degrading proteins, respectively. Other CBMs detected in this study were unrelated to polysaccharide degradation. Given the types of CBMs detected in the yak rumen and the scarcity of CBMs in proteins targeting plant cell walls, the free-enzyme system may not be the predominant one in lignocelluloses degradation in yak rumen.

Some cellulolytic anaerobes produce cellulosomes, a multi-protein complex that is held together through the cohesin-dockerin interaction. The presence of the dockerin or cohesin module in a protein, therefore, suggests that the protein is a potential component of a cellulosome [7]. 516 and 51 sequences were found to encode putative dockerin and cohesin modules, respectively, in the metagenome of yak rumen microbiota. However, none of the GH family proteins retrieved from the active BAC clones contained such modules (Table S2). Similar observations were made in other studies of ruminants and herbivores [5], [11]. Dockerin and cohesin detected in these microbiomes were in non-fibrolytic proteins [16]. Hence, fibrolysis by cellulosome-like cellulolytic protein complexes was unlikely the predominant mechanism in plant cell wall degradation in the yak rumen. Taken together, our data suggest that the yak rumen microbiome may employ a mechanism involving the use of SusC and SusD-like membrane proteins in plant cell wall hydrolysis.

### Identification and Biochemical Characterization of Two Proteins of a Novel GH5 Subfamily

**Figure 3:**
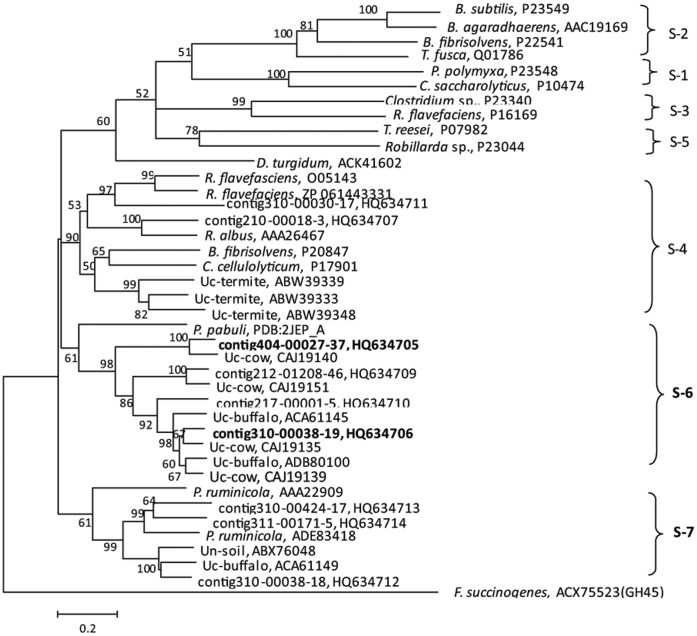
Phylogenetic tree of the putative GH5 proteins encoded by genes from the BAC library. The tree was constructed from 300 amino acid sequences using Mage 3.0 software. ACX75523 (GH45), an endoglucanase from *Fibrobacter succinogenes*, was used as the outgroup. Names of the organisms from which sequences are derived are given. Sequences with an “Uc” prefix refer to uncultured clones, and those with “contig” are from this study. Proteins with sequences in boldface were overexpressed and characterized in this study. Cluster affiliation of glycoside hydrolase families are given on the right, and the GenBank accession numbers follow the sequence names. Numbers at the cluster nodes are the supporting percentages of bootstrap evaluation. Bar, 20% sequence divergence.

As mentioned above, while genes encoding cellobiohydrolases or proteins of the GH48 family were barely detected, GH5 proteins represented the most diverse and predominant group of the cellulases in the yak rumen microbiome (Table 1 and Table S3). In order to understand the mechanism of crystalline cellulose hydrolysis in the exocellulase-scarce rumen, all GH5 proteins retrieved from the BAC clone library were analyzed. The GH5 cellulases are currently divided into five subfamilies on the basis of their amino acid sequence similarity [17]. Phylogenetic analysis shows that the GH5 proteins identified in the present study fall into three clusters. One of the clusters belongs to subfamily 4, which is found primarily in the rumen and termite gut; the other two branch off from the described five GH5 subfamilies, and are tentatively designated as GH5 subfamily 6 and 7 (Figure 3). GH5 subfamily 6 includes members found exclusively in uncultured rumen microorganisms. These genes encode proteins that share 26–30% amino acid sequence identities with a xyloglucanase from *Paenibacillus pabuli* [18]. GH5 subfamily 7 consists of genes from *Prevotella ruminicola* and uncultured rumen and soil microorganisms [19].

To determine the enzymatic activities of the novel GH5 subfamily 6 proteins, whose encoding genes occurred 5–10 fold more frequently than an average gene in the metagenome, two ORFs (contig404-00027-37 and contig310-00038-19) from the yak rumen clones were overexpressed in *E. coli*. Both recombinant proteins were active on mannose, birch wood and carboxylmethyl cellulose (CMC) (Table S5). Intriguingly, the two proteins showed low but measurable hydrolytic activity (0.16 and 0.055 U/mg) on Avicel crystalline cellulose (Figure S3), suggesting that they may serve as an exoglucanase-like activity in the yak rumen.

## Discussion

Because of their high efficiency in plant cell-wall degradation, rumen microbiomes have attracted enormous research attention over the past few decades. The complex lignocellulase systems and their synergistic actions underpinning the robust lignocellulose hydrolysis in the rumen have presented a challenge to our understanding. Metagenomic approaches permit analysis of the rumen microbiome at a level of complexity that has never been achieved previously. In the present work, by combining the pyrosequencing of the metagenomic DNA and fibrolytic active BAC clones prepared with the same DNA pool, we gained insight into the profile of the fibrolytic genes and the organizational patterns of fibrolytic gene clusters that are indicative of lignocellulose degradation mechanisms in the yak rumen. As the long, high-quality contig sequences for the functional BAC clones gave high-resolution details of the gene and operon structures, such genotype-phenotype integrated screening approaches allowed in-depth analysis of the organizational patterns of fibrolytic genes in the genomes. The latter knowledge is of major value in understanding the coordination and regulation of the expression of fibrolytic genes, especially those grouped in clusters.

The majority of the GH proteins, especially those situated in the large contigs retrieved from the BAC library, were found from the Bacteroidetes and Firmicutes, with fewer from the Fibrobacteres. This implies that, similar to the situation in other rumen and herbivorous gut microbiomes [5], [11], the Bacteroidetes and Firmicutes likely play a major role in plant cell-wall degradation in the yak rumen.

Intriguingly, fibrolytic genes were linked to genes encoding the SusC/SusD-like proteins on 50% of the Bacteroidetes-derived contigs. SusC and SusD are outer-membrane proteins in the starch utilization system employed by human intestinal *Bacteroides* strains [9], [20], [21], [22], [23], they have also been found in other members of Cytophaga-Flavobacterium-Bacteroides (CFB), including *Cytophaga hutchinsonii*, an aerobic cellulolytic gliding bacterium [24], and *Flavobacterium johnsoniae* [25], a polysaccharide-digesting gliding bacterium. Consequently, their role in lignocellulose degradation has been proposed [25], [26]. Recently, based on the analysis of the genome sequences of several members of xylanolytic Bacteroidetes, the Sus-like genes have been speculated to be involved in xylan utilization by gut Bacteroidetes strains [26].

Similar to those from Bacteroidetes, the majority of the GH proteins derived from Firmicutes contain a signal peptide, but no CBM. Likewise, our data do not provide evidence for the use of a free-enzyme system by fibrolytic Firmicutes in the yak rumen. *Fibrobacter succinogenes* derived fibrolytic contigs seem not to dominant in the yak rumen. Thus, the Fibrobacter-specific fibro-slime protein-assisted cellulose deconstruction model, which has been proposed based on the recent *F. succinogenes* genome data [4], is probably not the predominant mechanism in the yak rumen. Based on the collective data from domain architectures of the GH proteins targeting plant cell walls, and the characteristics of the gene clusters relevant to fibrolysis, we propose that the SusC/SusD-mediated lignocellulose degradation is responsible for the highly efficient degradation of lignocellulose in the yak rumen microbiome.

GH 48 genes are rarely found in the yak rumen microbiome, as reported in previous studies on other rumens [5], [6]. This raises the possibility that other proteins display exo-type activities in cellulose hydrolysis in the rumen. The two GH5 enzymes obtained from the yak rumen are potential candidates for their weak but detectable avicelase activity. GH5 proteins have long been known to possess activities of endoglucanase, endoxylanase, mannanase, etc. However, it was recently reported that a GH5 protein from a marine bacterium [27] and a cellulase from buffalo rumens, which has been identified as a member of the subfamily 7 of GH5 in this study, showed exoglucanase activity [19]. Taken together, these findings suggest that the rumen microbiome may employ a previously unknown strategy for lignocellulose degradation.

## Materials and Methods

### Rumen Sampling

A mixture of rumen fluid and undigested fiber was taken from two Qinghai-Tibetan domesticated yaks fitted with rumen fistula (each ∼500 kg in body weight), which had been fed on a wheat stalk diet for two weeks. All animal procedures were approved by the Committee on the Ethics of Animal Experiments of the Institute of Microbiology, Chinese Academy of Sciences, China (permit number: PZIMCAS2008001) and all efforts were made to minimize suffering of the animals. Two rumen sample (∼100 ml each) were centrifuged at 30,000 g at 4°C for 30 min and then were mixed and stored at –70°C before use. Additional details are provided in the **information S1.**

### DNA Extraction

Genomic DNA was extracted from the rumen sample and purified according to the protocol of Walter [28] with modifications. Briefly, a cell pellet embedded in low-melting-point agarose was immersed in a lysis solution with HindIII for 20 min at 37°C. The DNA in the agarose plug was then used for metagenomic sequencing. To construct the BAC library, the agarose plug with DNA was subjected to pulsed-field gel electrophoresis using the CHEF Mapper System (Bio-Rad, Hercules, California), and 50- to 200-kb fragments were recovered from the gel by electroelution. Additional details are provided in the **information S1.**

### BAC Library Construction

A sample (100 ng) of the HindIII-digested DNA fragment was ligated into copy-control plasmid pCC1BAC Cloning-Ready Vector (25 ng; Epicentre, Madison, WI), which had been cleaved with HindIII, according to the manufacturer’s instructions. The ligation mixture (2 µl) was electroporated into *Escherichia coli* EPI300 electro-competent cells (20 µl, Epicentre) using the GenePulser Xcell (Bio-Rad) as described previously [29]. The transformed cells were immediately inoculated into ice-cold SOC medium (0.5 ml) and allowed to recover at 37°C for 1 h before plating. After incubation at 37°C for 16 h, white colonies were picked using the QPix2 XT robotic colony-picking workstation (Genetix, New Milton, Hampshire, UK) and inoculated into 384-well microtiter plates containing LB medium with 12.5 µg/ml chloramphenicol and 10% (v/v) glycerol. The clones were stored at –80°C.

### Screening of Fibrolytic BAC Clones Based on Enzyme Activities

Colonies in 384-well plate were transferred to large Petri dishes containing LB with chloromphenicol and various substrates corresponding to screening cellulase, xylanase, esterase, and lipase activities, the key enzymes in lignocelluloses degradation. Additional details are provided in the **information S1.**

### Sequencing and Assembly of the Cellulolytic BACs

A total of 223 BAC clones with fibrolytic activity was selected for sequencing. BAC clone DNA was extracted and purified using the QIAGEN Large-Construct kit (Qiagen, Hilden, Germany). Each pool of BAC clone DNA (typically including 20 BACs) was fragmented and ligated with a specific barcode and subjected to pyrosequencing on the 454 Life Sciences Genome Sequencer GS FLX Titanium (Roche, USA). A total of 838,584 reads, or 299.88 Mbp of DNA sequence, was generated. The sequences, with an average read length of 357 bp, were screened (using the bovine genome), trimmed (using the BAC vector sequence), and assembled using Newbler (version 2.3). In all, 4,936 contigs, with a total length of 14.19 Mb, were obtained. The N50 was 25,621 bp and the largest contig about 103 kb in length.

### Sequencing and Assembly of the Metagenome

The ruminal total genomic DNA was fragmented and ligated with the sequencing adaptors, then sequenced by the 454 GS-FLX Titanium. A total of 239,344 reads, amounting to 88 Mb data, was collected, with an average read length of 367 bp.

Total metagenomic DNA libraries were also prepared and sequenced on two lanes of pair-end flow-cells on the Solexa GA-IIx (Illumina, USA), in which 37,319,846 paired reads with a read length of 76 bp and 64,726,030 paired reads with a read length of 100 bp, respectively, were obtained.

The Solexa reads were then trimmed according to their quality and assembled using the VCAKE assembly pipeline (version 1.1). In all, 2,817,877 contigs, with a total base of 429 Mb, were obtained. The N50 of the assembly was 140 bp and the largest contig 18,366 bp. The generated contigs were chopped to simulate pyrosequencing shotgun reads (with a read length of 500 and 3 fold sequence coverage); paired-end reads (with different insert sizes: 2 k, 5 k, 7 k, 10 k, and 15 k, with a read length of 500 bp and threefold coverage) were then assembled using Newbler (version 2.3) together with the reads produced by the GS FLX Titanium. In all, 3,718 scaffolds with N50 of 3,596 bp were produced. The largest scaffold size was 24,412 bp.

### Coverage Analysis of BAC Sequences

All 2,817,877 metagenomic contigs generated from the VCAKE assembly pipeline as well as the 35 BAC-derived contigs were aligned against all the Solexa reads using Bowtie [30]. The best alignment for each read was retained which allows for up to 10% mismatch sites over the entire read length. The average sequence coverage for all the metagenomic contigs was calculated by the length of all aligned reads over the total contigs length. Individual sequence coverage of 35 contigs was calculated similarly, i.e. dividing the cumulative aligned reads length mapped to each contig by the contig length.

### Gene Prediction and Annotation

Open reading frames were predicted using MetaGeneAnnotator [31], [32]. Module and domain analysis was performed against Pfam24.0 database [10] using HMMER 3.0 [33]. The results were then parsed by perl scripts and stored into the MySQL database. The ORFs were categorized against the updated KEGG pathway database (ftp://ftp.genome.jp/pub/kegg/in 20100619) and COG database (http://www.ncbi.nlm.nih.gov/COG) using BLAST. Annotations were performed manually by combining the search results against the databases of nr, COG, KEGG, and Pfam. Secreted proteins were predicted using SignalP 3.0 (http://www.cbs.dtu.dk/services/SignalP/). Both neural networks and hidden Markov models were used by selecting the “Organism group” parameters for Gram-negative and positive bacteria.

Glycoside hydrolases (GH), and carbohydrate-binding modules (CBMs) were grouped based on gene annotation and analysis of conserved motifs [11], [12], [34] deposited in the Carbohydrate-Active enZYmes Database (CAZy; http://www.cazy.org).

### Phylogenetic Analysis

All of the lignocellulytic enzyme-encoding genes and the ORFs in 35 contigs were determined for their phylogenetic origin by MAGEN [35] and PhymmBL V3.1, which combined analysis from both Phymm and BLAST and could accurately classify DNA sequences as short as 100 bp [36].

Amino acid sequences of GH5 proteins from yak rumen and their homologs retrieved from GenBank (http://www.ncbi.nlm.nih.gov), UniProt (http://www.uniprot.org) and CAZy (http://www.cazy.org/GH5.html) were performed a multiple sequence alignments using Clustal X1.83. Phylogenetic trees were constructed using neighbor-joining method with MEGA 3 software package [37].

### Gene Cloning and Expression

Genes encoding two novel GH5 cellulases (contig404-00027-37 and contig310-00038-19) were amplified with PCR using a mixture of the BAC clone DNAs as template. The primer pairs for amplification of contig404-00027-37 were 5′-AACTCATATGATGAAATCCTATTATTATCAG- CTC-3′ and 5'-ATCTCGAGTTTAGGAGCACTGTTGTAAACAG-3′; those for contig310-00038-19 were 5′-GGAATTCCATATGAAAAGATACCTGACCC- TCCT-3′ and 5′-CCGCTCGAGGAACTTGGGGGCCGATTTATAG-3′. The NdeI site in the forward and XhoI site in the reverse primers are underlined. PCR products were cloned into the expression vector pET-30a(+) (Novagen) and transformed into *E. coli* Rosetta (DE3) pLysS (Novagen). The proteins were purified by affinity chromatography on nickel nitrilotriacetic acid agarose resin (Ni-NTA; GE healthcare, Sweden) according to the manufacturer’s instructions. Protein concentration was determined using the BCA Protein Assay Kit (Thermo Scientific, Rockford, US) with bovine serum albumin as the calibration standard. The methods for characterization of the purified proteins are provided in the **information S1.**

## Supporting Information

Figure S1 **Screening of fibrolytic BAC clones.** (A), cellulase activity screening using carboxymethyl cellulose (CMC) as the tested substrate and Congo red as the indicator; (B), birch wood xylan as the tested substrate and 3,5-Dinitrosalicylic acid (DNS) as the indicator; (C), esterase activity screening using α-naphthyl acetate as the indicator.
(DOC)

Figure S2 **Gene organizations and coverage of the ORFs on 35 fibrolytic contigs retrieved from BAC clone library.** Colored ORFs indicated the coverage over the average coverage of the metagenome reads. Blue, >100 fold higher; Red, 10–100 fold; Green, 8–10 fold; Purple, 2–8 fold; Sky-blue, 0.5–2 fold; Black, 0.25–0.5 fold; White, <0.25 fold. In the parenthesis refer to the activities on which the clones are screened. Scale labels the nucleotide base pair. (**A**), contigs affiliated to Bacteroidetes; (**B**), contigs affiliated to Firmicutes; (**C**), contigs affiliated to *Fibrobacter succinogens*; (**D**), contigs affiliated to other phyla of bacteria.
(DOC)

Figure S3 **Exocellulase activity assay of the recombinant protein of ORF 310-00038-19 using filter paper (panel A) and Avicle (panel B) as the substrate.**
(DOC)

Table S1 **Summary of the sequencing data both of metagenome and 223 BAC clones showing various fibrolytic activities from yak rumen.**
(DOC)

Table S2 **Domain architectures of the putative cellulolytic glycosidases retrieved from the BAC expression library constructed for yak rumen microbiome and their closeted relatives.**
(DOC)

Table S3 **GH and CBM profiles determined in the BAC library and metagenome of yak rumen microbiome.**
(DOC)

Table S4 **Identified enzymatic activities of the proteins from various glycoside hydrolase families those targeting plant-cell wall in Carbohydrate-Active enZYmes Database (CAZy).**
(DOC)

Table S5 **Cellulase and hemicellulase activities of the over expressed proteins from the novel subfamily 6 of GH5 obtained in the yak rumen.**
(DOC)

Information S1 **Supplementary Methods.**
(DOC)
