# Supplementary figures and images for: Metagenomic Insights into the Fibrolytic Microbiome in Yak Rumen
Source: PLoS One. 2012 Jul 13;7(7):e40430. doi: 10.1371/journal.pone.0040430 (PMC3396655; doi:10.1371/journal.pone.0040430)

**Fig. S1.**


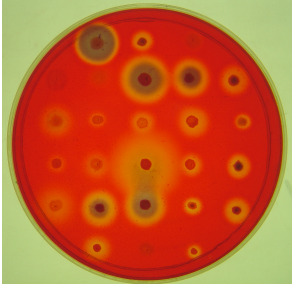


A


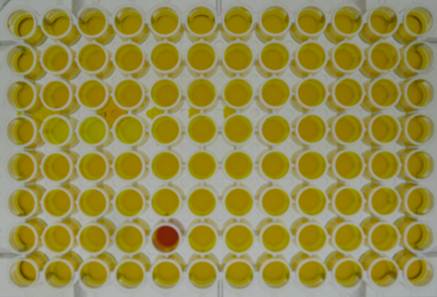


B


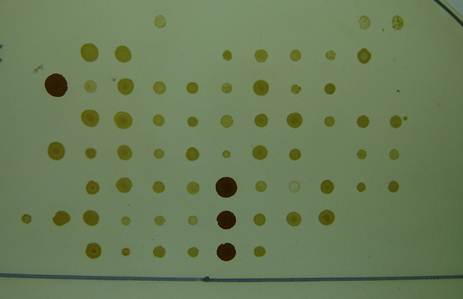


C

Supplement: Figure S1 — Screening of fibrolytic BAC clones. (A), cellulase activity screening using carboxymethyl cellulose (CMC) as the tested substrate and Congo red as the indicator; (B), birch wood xylan as the tested substrate and 3,5-Dinitrosalicylic acid (DNS) as the indicator; (C), esterase activity screening using α-naphthyl acetate as the indicator. (DOC) [file pone.0040430.s001.doc]

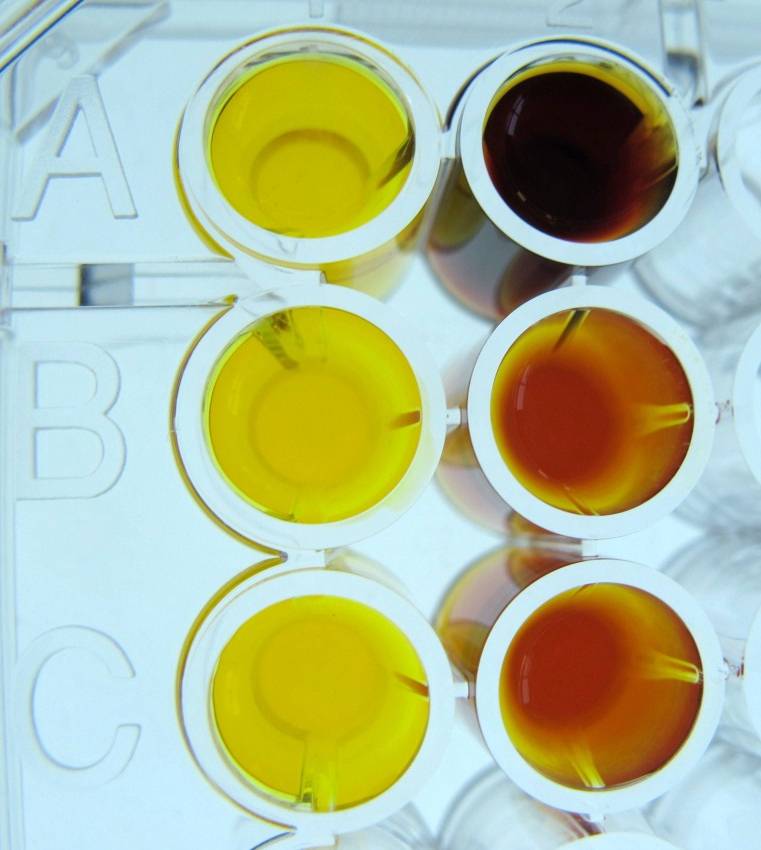
Fig S3

----

Contig310-00038-19 protein

A

B

+

Supplement: Figure S3 — Exocellulase activity assay of the recombinant protein of ORF 310-00038-19 using filter paper (panel A) and Avicle (panel B) as the substrate. (DOC) [file pone.0040430.s003.doc]
