# Supplementary material for: Metagenomic Insights into the Fibrolytic Microbiome in Yak Rumen
Source: PLoS One. 2012 Jul 13;7(7):e40430. doi: 10.1371/journal.pone.0040430 (PMC3396655; doi:10.1371/journal.pone.0040430)

FigS2 Gene organizations on the contigs with fibrolytic enzymes retrieved from yak rumen microbiome via construction of BAC expression library. (35 fibrolytic degradation and 2 starch degradations.

A. Bacteroidetes:


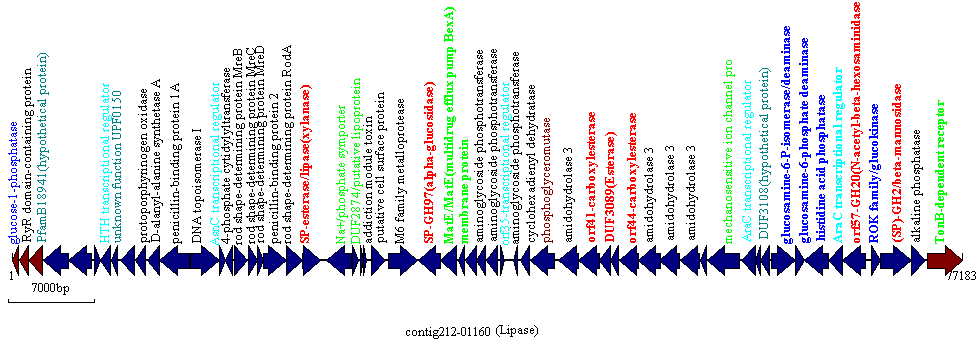


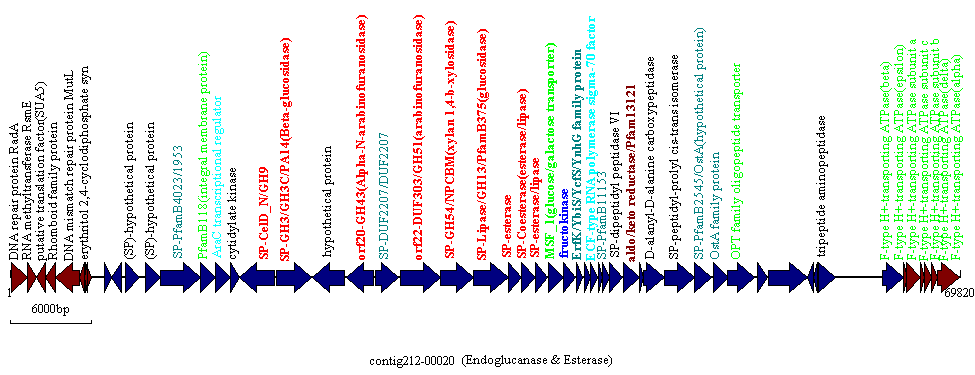


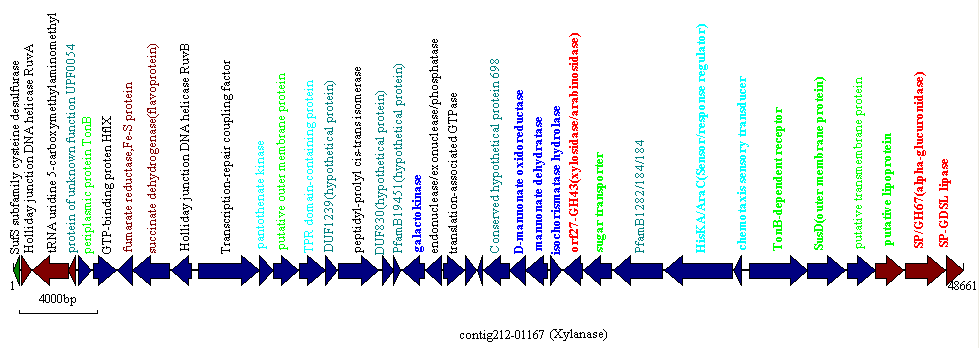


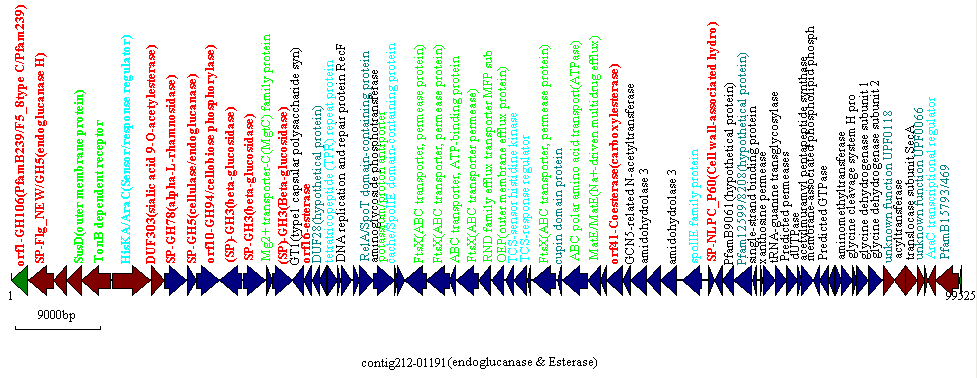


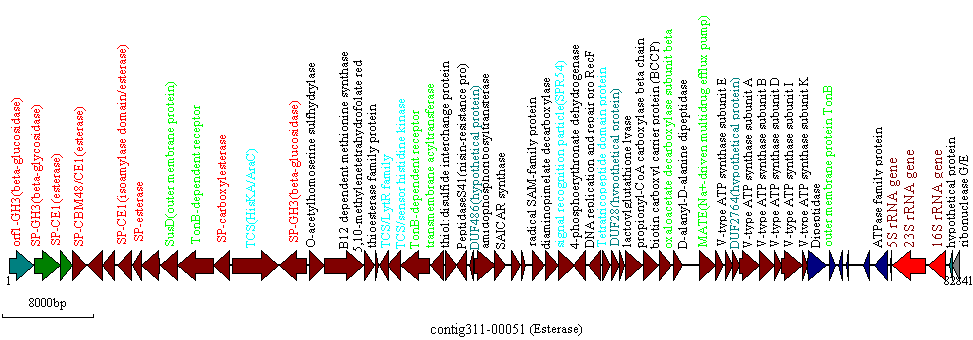


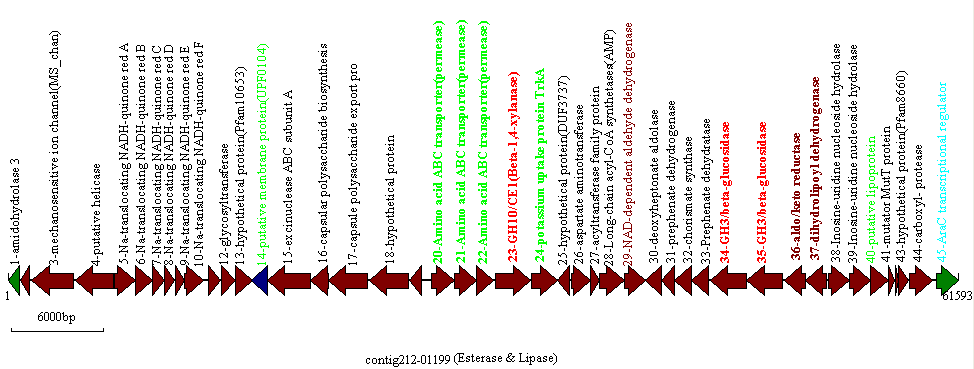


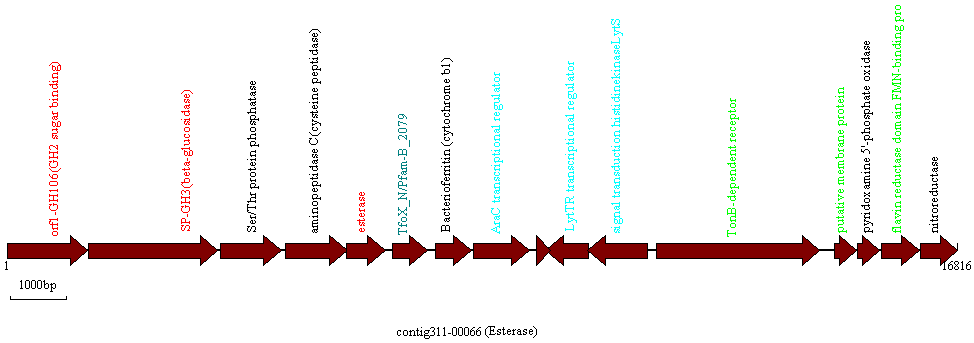


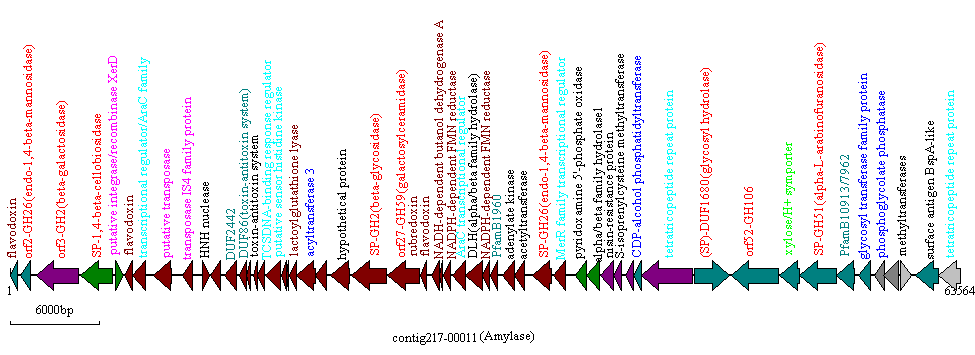


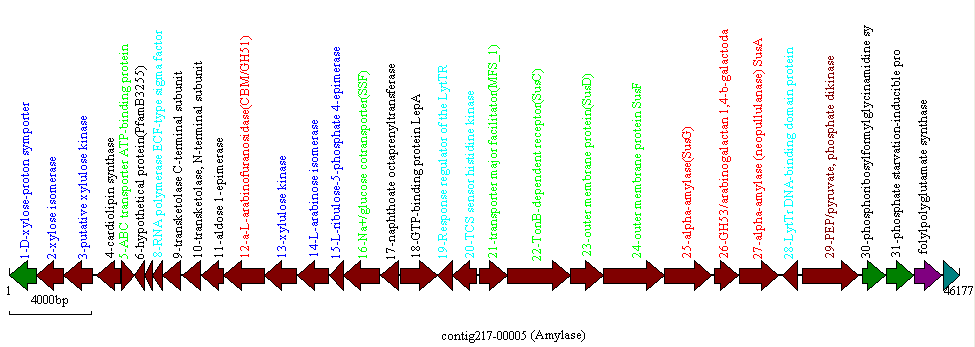


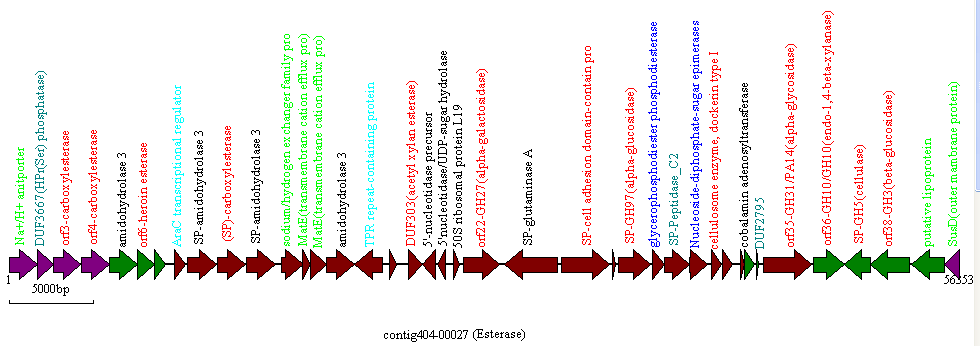


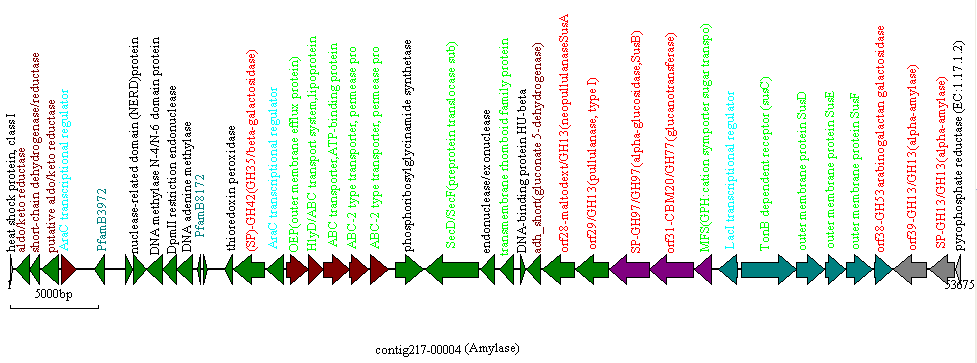


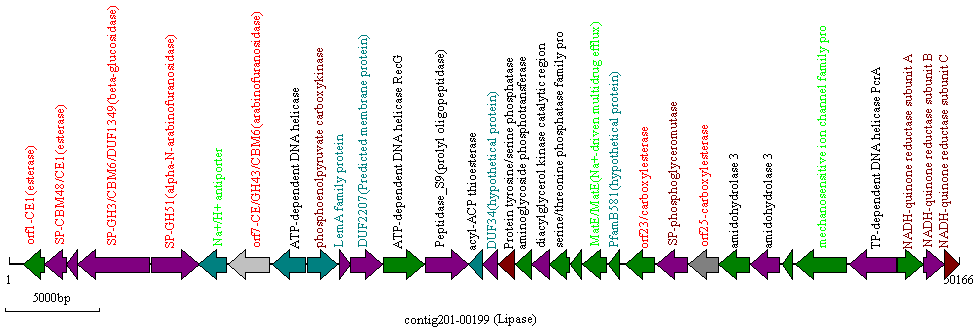


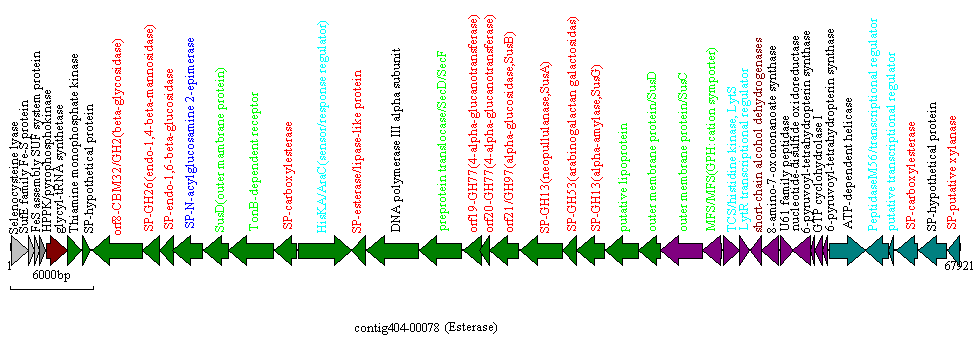


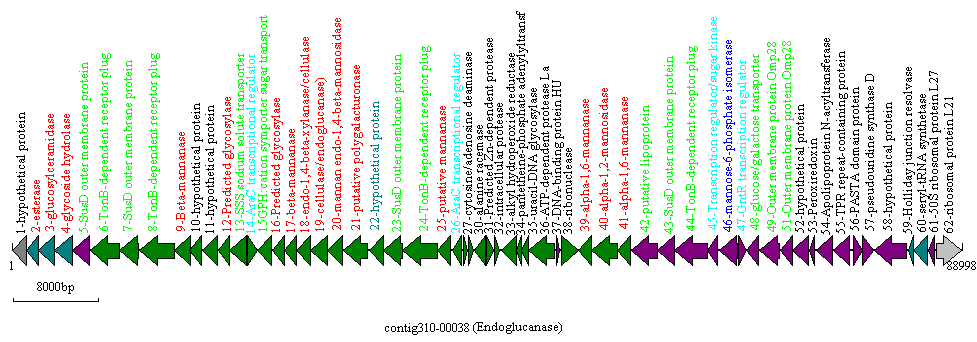


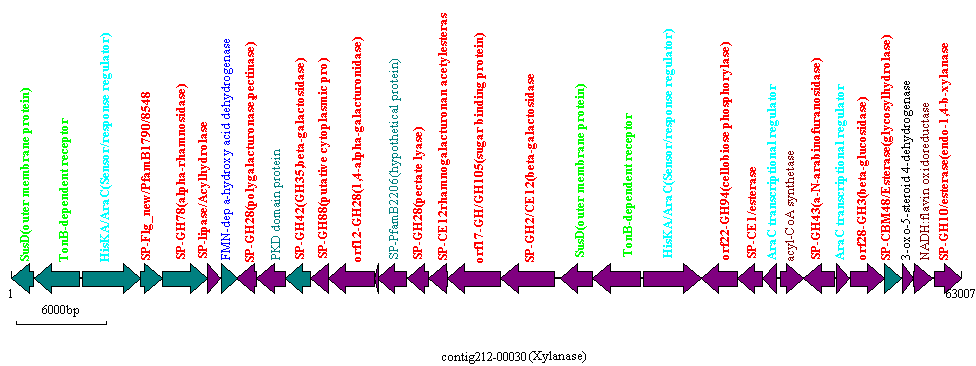


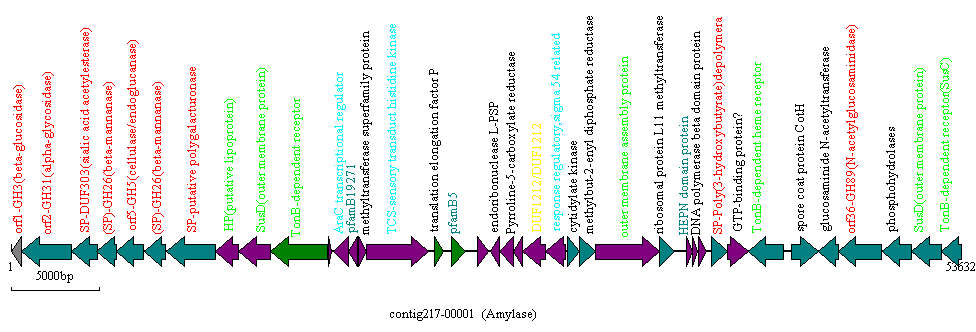


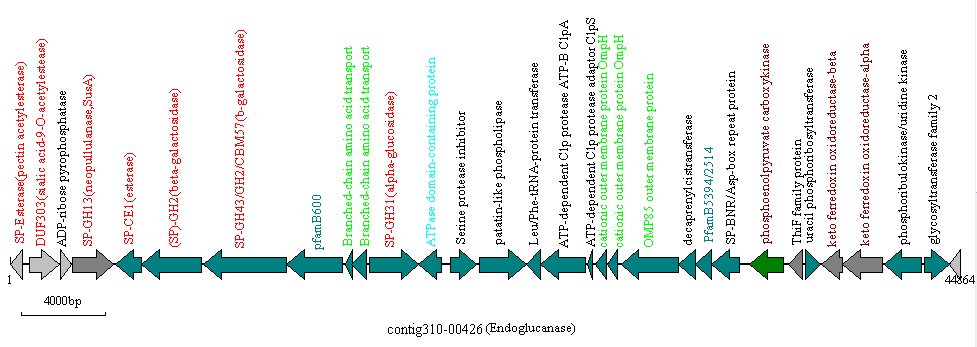


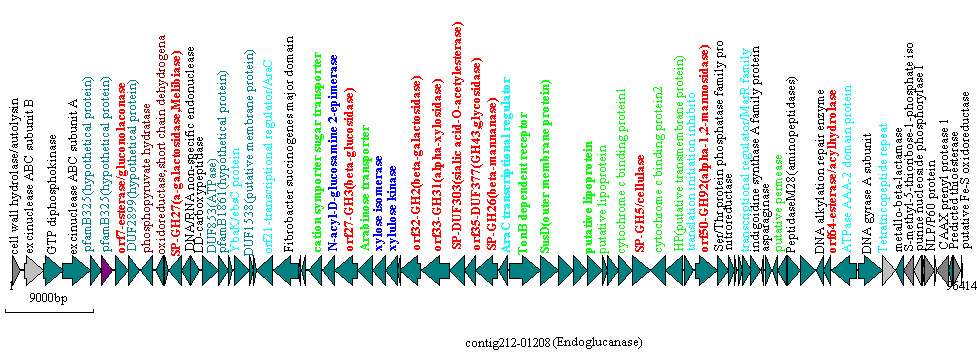


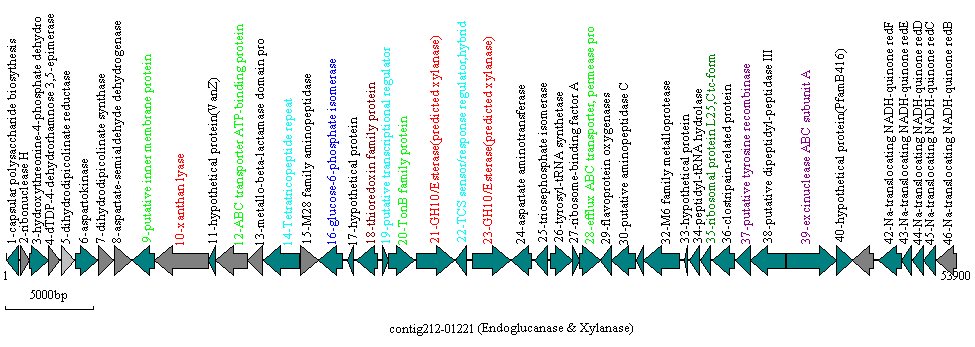


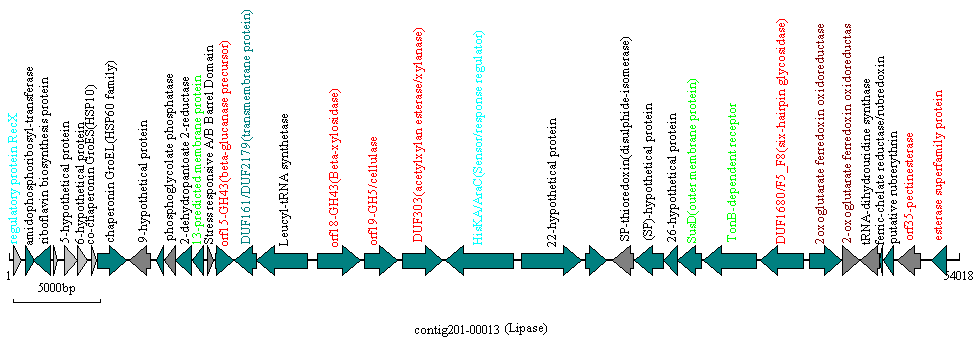


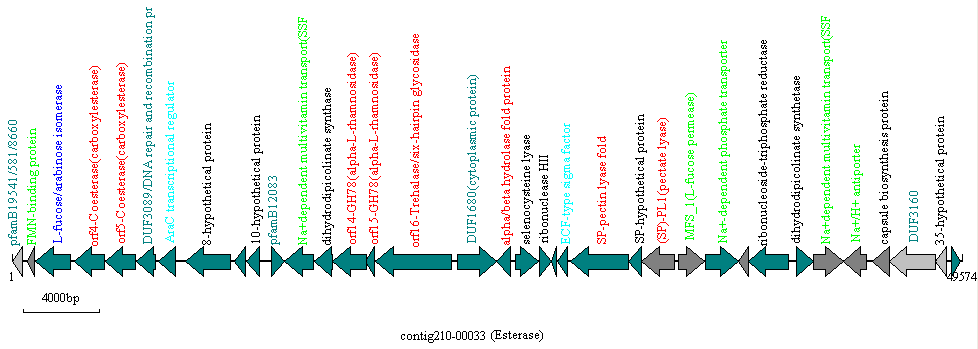


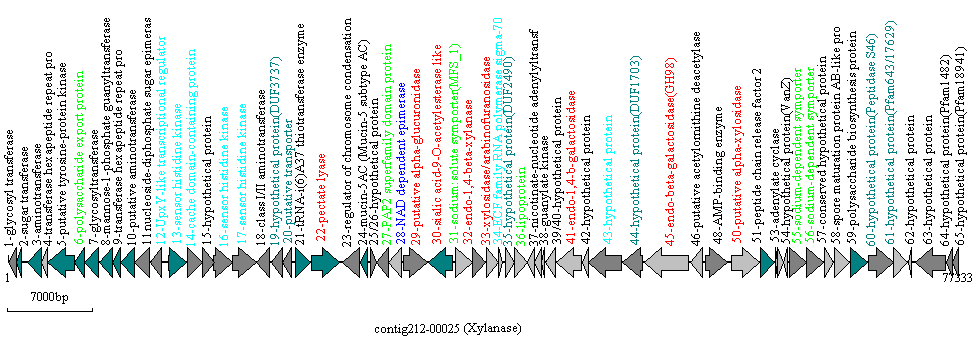


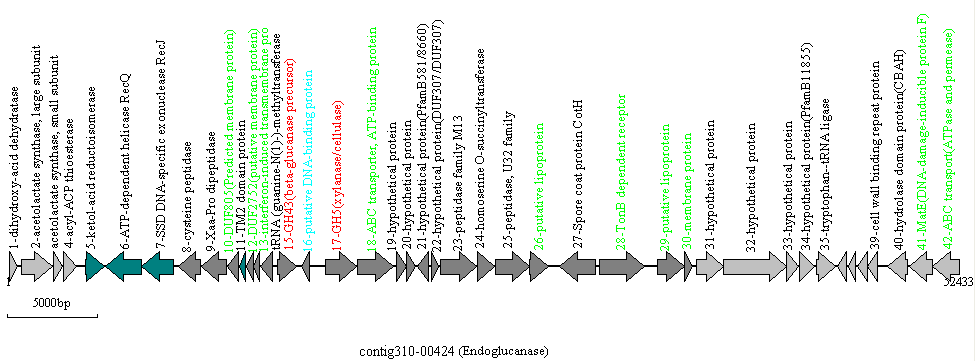


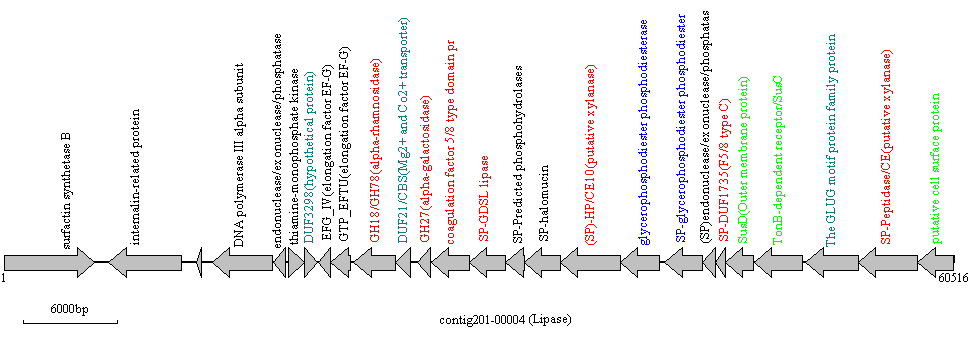


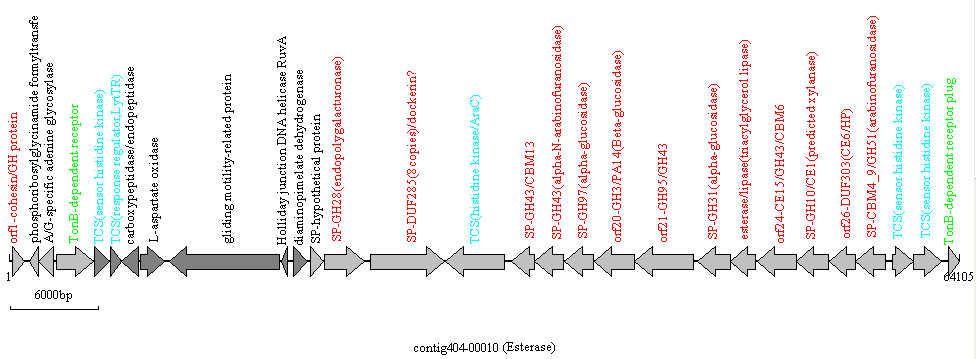


B. Firmicutes:


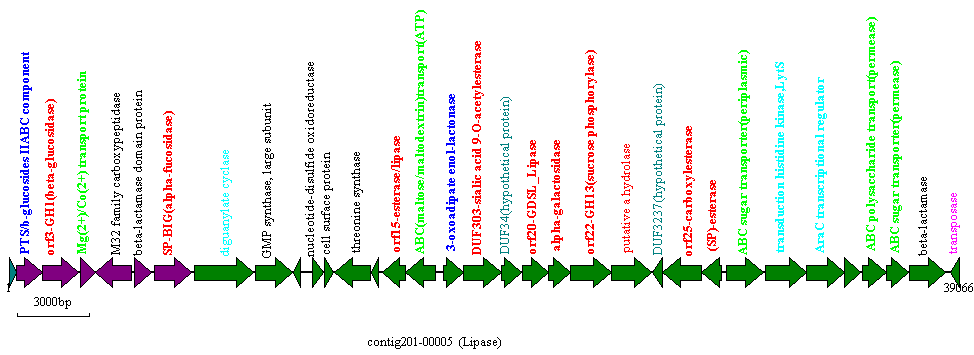


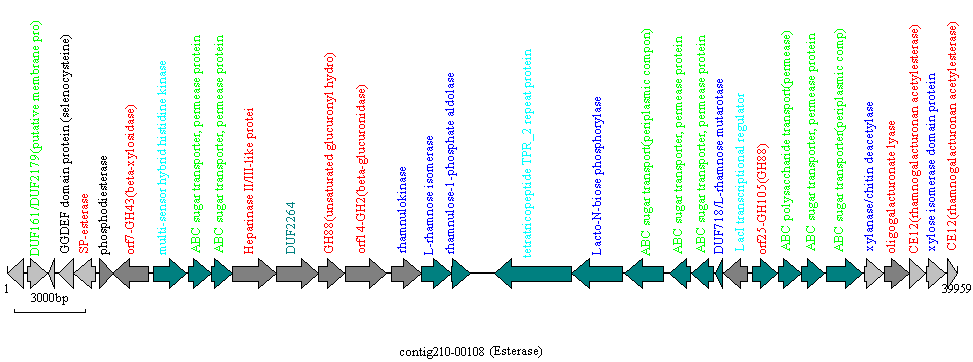


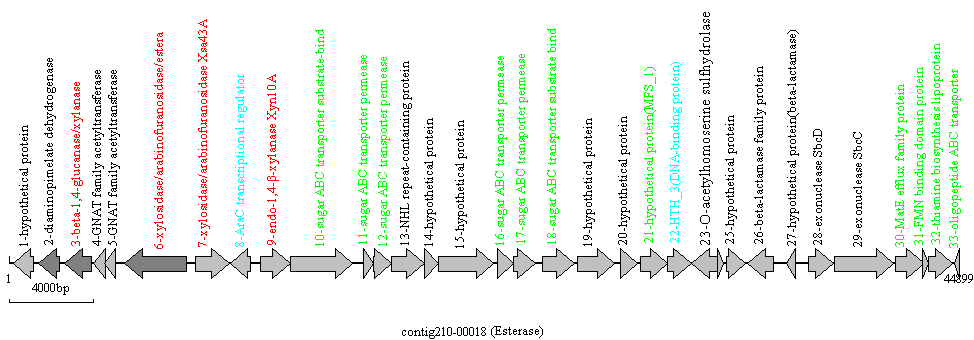


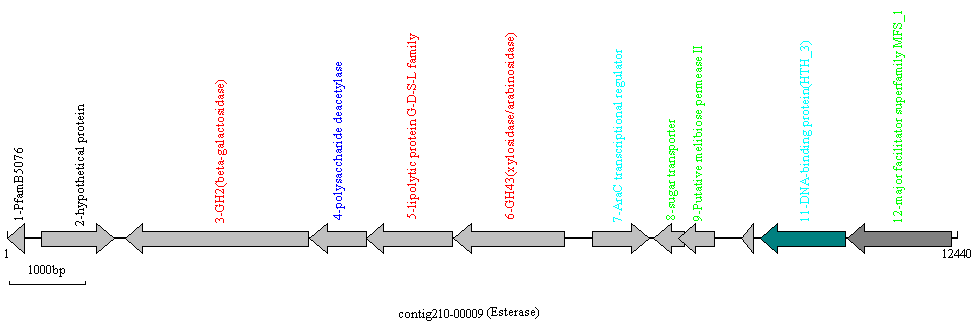


C. Fibrobacter:


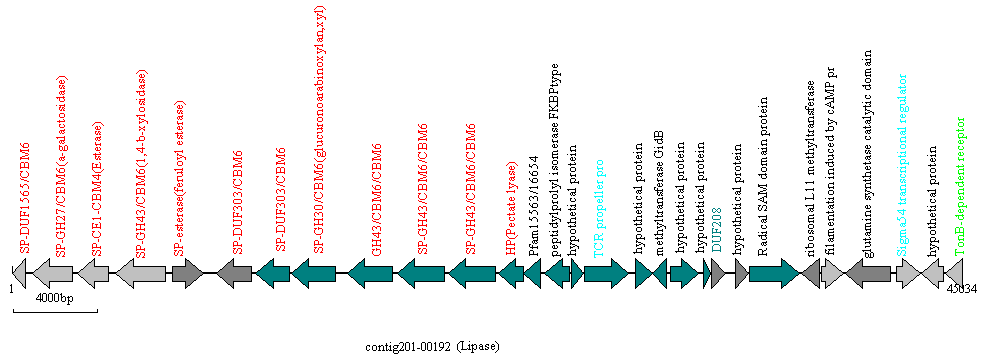


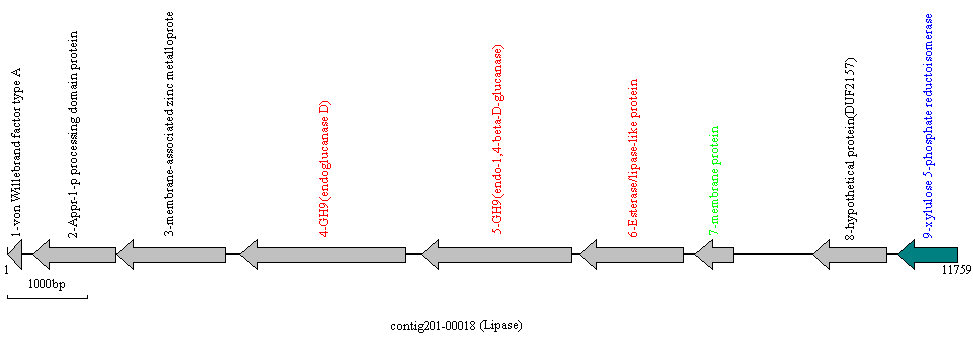


D. Unknown:


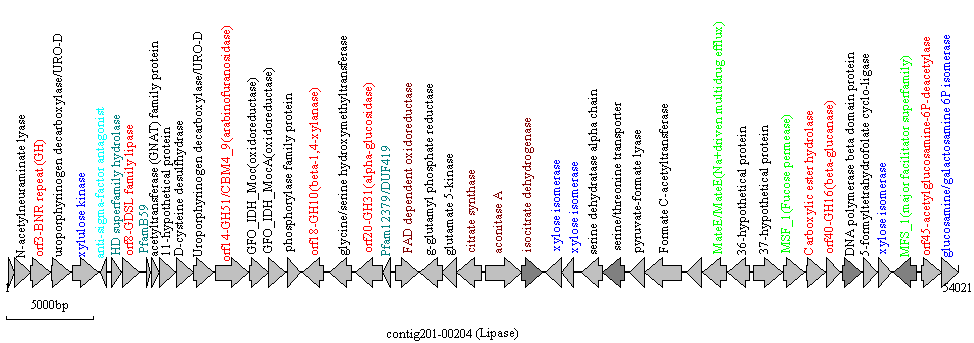


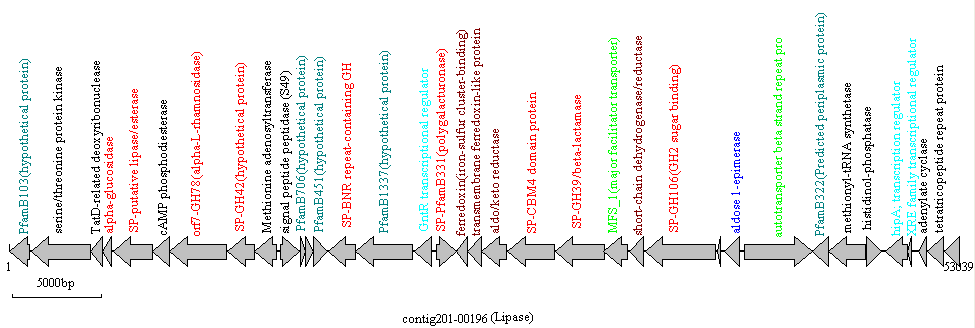


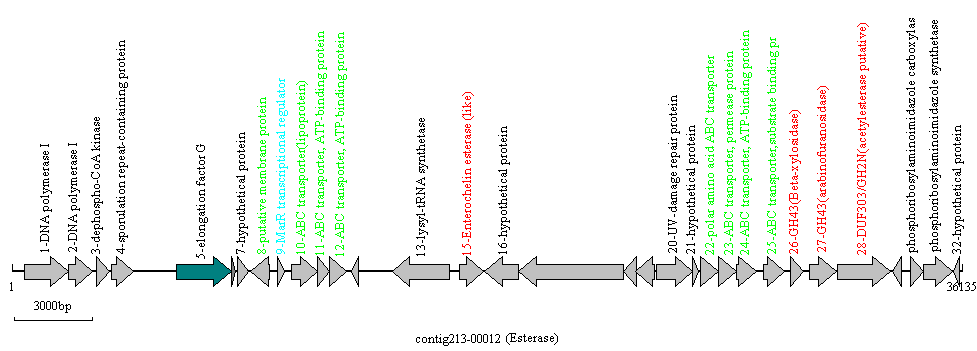


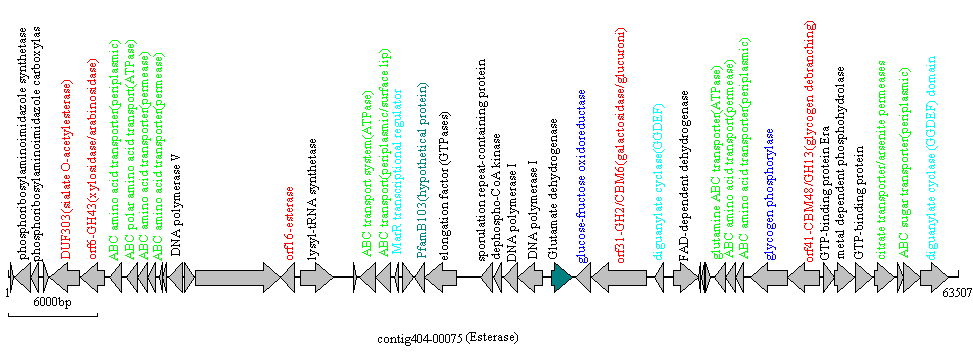

Supplement: Figure S2 — Gene organizations and coverage of the ORFs on 35 fibrolytic contigs retrieved from BAC clone library. Colored ORFs indicated the coverage over the average coverage of the metagenome reads. Blue, >100 fold higher; Red, 10–100 fold; Green, 8–10 fold; Purple, 2–8 fold; Sky-blue, 0.5–2 fold; Black, 0.25–0.5 fold; White, <0.25 fold. In the parenthesis refer to the activities on which the clones are screened. Scale labels the nucleotide base pair. (A), contigs affiliated to Bacteroidetes; (B), contigs affiliated to Firmicutes; (C), contigs affiliated to Fibrobacter succinogens; (D), contigs affiliated to other phyla of bacteria. (DOC) [file pone.0040430.s002.doc]
