# Supplementary material for: Metagenomic Insights into the Fibrolytic Microbiome in Yak Rumen
Source: PLoS One. 2012 Jul 13;7(7):e40430. doi: 10.1371/journal.pone.0040430 (PMC3396655; doi:10.1371/journal.pone.0040430)
Supplement: Table S1 — Summary of the sequencing data both of metagenome and 223 BAC clones showing various fibrolytic activities from yak rumen. (DOC) [file pone.0040430.s004.doc]

**Table S1. Summary of the sequencing data both of metagenome and 223 BAC clones showing various fibrolytic activities from yak rumen**

|  | | BAC | Metagenome | |
| --- | --- | --- | --- | --- |
|  |  | 454 | 454 | Solexa |
| Sequencing & assembly | Number of reads | 838,584 | 239,344 | 37,319,846 (2*76)  64,726,030 (2*100) |
|  | Avg. length (bp) | 357.61 | 367.70 | - |
|  | Total sequences  ( bp ) | 299,884,856 | 88,007,868 | 9,308,911,296 |
|  | Number of scaffolds | - | 3,718 | |
|  | Total bases of  scaffolds (bp) | - | 13,307,418 | |
|  | N50 | 25,621 | 3,596 (scaffold)  1,042 (contig) | |
|  | Assembled contigs (>500bp) | 2,310 | 5,134 | |
|  | Contig size (bp) | 13,229,749 | 51,728,319 | |
|  | Coverage (fold) | 21.14 | - | |
|  | Largest contigs (bp) | 103,222 | 24,412 | |
| ORF predicted | | 10,070* | 7,758,816^*^ | |
| Putative fibrolytic genes | scattered | 47 | 19,527 | |
|  | Clustered or linked | 103 |  |  |

*, Complete orfs are predicted based on MetaGeneAnnotator program.
