# Supplementary material for: Metagenomic Insights into the Fibrolytic Microbiome in Yak Rumen
Source: PLoS One. 2012 Jul 13;7(7):e40430. doi: 10.1371/journal.pone.0040430 (PMC3396655; doi:10.1371/journal.pone.0040430)
Supplement: Table S2 — Domain architectures of the putative cellulolytic glycosidases retrieved from the BAC expression library constructed for yak rumen microbiome and their closeted relatives. (DOC) [file pone.0040430.s005.doc]

**Table S2. Domain architectures of the putative cellulolytic glycosidases retrieved from the BAC expression library constructed for yak rumen microbiome and their closeted relatives ^1^**

| Orfs | Pfam | aa | Most closed relatives (organisms) | Phylum | E value | Identity (%) |
| --- | --- | --- | --- | --- | --- | --- |
| Contig217-00001-5 | SP-GH5 | 506 | Cellulase/endoglucanase (unidentified microorganism, CAJ19135)  Hypothetical protein (*Bacteroides cellulosilyticus*, EEF89776) | B | 1e-159  1e-104 | 298/528=56  219/471=46 |
| contig404-00027-37 | SP-GH5 | 514 | Cellulase (unidentified microorganism, CAJ19140)  Hypothetical protein (*Bacteroides cellulosilyticus*, EEF89776) | B | 0.0  1e-103 | 359/519=69%  216/467=46% |
| contig210-00018-3 | SP-GH5 | 417 | endo-1,4-beta-glucanase/xylanase Cel5A (*Butyrivibrio proteoclasticus* B316, ADL33047) | F | 0.0 | 407/440=92% |
| contig310-00030-17 | SP-GH5-P | 722 | Endoglucanase A (*Ruminococcus flavefaciens*, ZP_06144331) | F | 1e-112 | 217/517=41% |
| contig310-00038-4 | SP-GH5 | 544 | glycoside hydrolase (*Zunongwangia profunda* SM-A87, ADF53794) | B | 1e-153 | 248/461=53% |
| contig310-00038-18 | SP-GH5 | 428 | Cellulase (uncultured microorganism, ACA61149)  Xylanase (*Prevotella ruminicola*, AAC36862) | B | 1e-171  2e-97 | 292/401=72%  191/336=48% |
| contig310-00038-19 | (SP)-GH5 | 526 | endoglycosidase precursor protein (uncultured bacterium, ABB46200 )  B-1,4-endoglucanase (*Prevotella bryantii*, AAC97596) | B | 1e-121  1e-121 | 215/337=63%  201/344=58% |
| contig310-00424-17 | SP-GH5 | 575 | Xylanase (*Prevotella ruminicola*, AAC36862) | B | 0.0 | 351/585=60% |
| contig311-00171-5 | GH5 | 534 | Endo-1,4-β-D-glucanase(unidentified microorganism, ABX76045)  Xylanase (*Prevotella ruminicola*, AAC36862) | B | 0.0  1e-139 | 325/487=66%  264/517=51% |
| contig212-01191-2 | SP-Flg_new-P- (P-P)-GH5 | 853 | Hypothetical protein (*Bacteroides uniformis*, EDO52405) | B | 6e-96 | 186/450=41% |
| contig212-01191-9 | SP-GH5 | 320 | Hypothetical protein (*Bacteroides uniformis*, EDO55904) | B | 7e-95 | 166/325=51% |
| contig212-01208-46 | SP-GH5 | 590 | Cellulase (unidentified microorganism, CAJ19151)  Hypothetical protein (*Bacteroides cellulosilyticus*, EEF89776) | B | 0.0  1e-121 | 361/484=74%  249/599=41% |
| contig310-00074-9 | SP-GH9 | 591 | glycoside hydrolase family 9 (*Fibrobacter succinogenes*, ACX75948) | Fib | 0.0 | 390/606=64% |
| contig212-00020-17 | SP-CelD_N-GH9 | 869 | glycoside hydrolase family 9 (*Paludibacter propionicigenes* WB4,YP_004041769) | B | 0.0 | 382/870=43% |
| contig406-00069-1 | SP-CelD_N-GH9 | 70 | endo-1,4-beta-D-glucanase (*Fibrobacter succinogenes*, AAC41523) | Fib | 1e-28 | 60/70=85% |
| contig310-00423-8 | **SP-CBM4_9-CelD_N-GH9** | 784 | hypothetical protein (*Coprococcus eutactus* ATCC 27759, EDP26786) | F | 0.0 | 362/790=45% |
| contig406-00006-1 | SP-CelD_N-GH9 | 237 | endoglucanase 1 (*Fibrobacter succinogenes*, ABU45498) | Fib | 1e-111 | 192/234=82% |
| contig201-00018-4 | SP-CelD_N-GH9 | 686 | glycoside hydrolase family 9 (*Fibrobacter succinogenes*, ACX75452) | Fib | 0.0 | 567/685=82% |
| contig201-00018-5 | SP-CelD_N-GH9 | 620 | endo-1,4-beta-D-glucanase (*Fibrobacter succinogenes,*AAC41523) | Fib | 0.0 | 489/618=79% |
| contig310-00427-5 | SP-GH45 | 400 | Cellulase (*Fibrobacter succinogenes*, ACX75523) | Fib | 0.0 | 363/390=93% |
| contig310-00014-4 | SP-GH8 | 735 | Glycoside hydrolase 8 (*Fibrobacter succinogenes*, ACX75395) | Fib | 0.0 | 467/723=64% |
| contig310-00015-1 | SP-GH8 | 366 | Glycoside hydrolase 8 (*Fibrobacter succinogenes*, ACX75395) | Fib | 0.0 | 288/369=78% |
| contig310-00421-3 | SP-GH8 | 735 | Glycoside hydrolase 8 (*Fibrobacter succinogenes*, ACX75395) | Fib | 0.0 | 612/737=83% |
| contig310-00429-2 | SP-GH8 | 336 | Glycoside hydrolase 8 (*Fibrobacter succinogenes*, ACX75395) | Fib | 0.0 | 271/339=79% |
| contig310-00433-3 | SP-GH8 | 555 | Glycoside hydrolase 8 (*Fibrobacter succinogenes*, ACX75395) | Fib | 0.0 | 341/545=62% |
| contig310-00434-16 | SP-GH8 | 479 | Glycoside hydrolase 8 (*Fibrobacter succinogenes*, ACX76164) | Fib | 0.0 | 431/481=89% |
| contig201-00201-18 | GH10-CE | 688 | β-1,4-D-xylanase (*Butyrivibrio fibrisolvens*,CAA43712) | F | 0.0 | 512/635=80% |
| contig201-00204-18 | GH10 | 408 | Glycoside hydrolase 10 (*Thermoanaerobcterium thermosaccharolyticum*, EET53393) | F | 1e-134 | 226/404=55% |
| contig201-00206-6 | SP-GH10-GH10 | 752 | Glycoside hydrolase 10 (*Fibrobacter succinogenes*,ACX75889) | Fib | 0.0 | 587/764=76% |
| contig404-00010-25 | SP-GH10-P-CE | 732 | xylanase/ferulic acid esterase (*Prevotella ruminicola*, ACN78954) | B | 0/0 | 609/730=83% |
| contig404-00027-36 | GH10-GH10 | 611 | Glycoside hydrolase 10 (*Prevotella ruminicola*,ADE83221) | B | 1e-115 | 208/335=62% |
| contig210-00018-9 | GH10 | 680 | Xylanase (*Eubacterium ruminntium*,BAA09971) | F | 1e-124 | 220/405=54% |
| contig310-00036-27 | SP-GH10-GH10 | 767 | Glycoside hydrolase 10 (*Fibrobacter succinogenes*,ACX75889) | Fib | 0.0 | 475/783=60% |
| contig310-00178-1 | GH10 | 134 | Hypothetical protein (*Bacteroides cellulosilyticus*,EEF88944) | B | 5e-21 | 56/129=43% |
| contig310-00210-2_ | SP-GH10 | 211 | Glycoside hydrolase 10 (*Bacteroides ovatus*,EFF52889) | B | 4e-10 | 33/93=35% |
| contig212-00025-32 | (SP)-GH10 | 363 | Glycoside hydrolase 10 (*Prevotella buccae*,EFC76128) | B | 1e-120 | 199/345=57% |
| contig212-00030-32 | SP-GH10-CE | 588 | Hypothetical protein (*Bacteroides eggerthii*,EEC53451) | B | 0.0 | 404/575=70% |
| contig212-01199-23 | SP-GH10-P-CE | 754 | Hypothetical protein (*Bacteroides eggerthii*,EEC53451) | B | 0.0 | 481/755=63% |
| contig212-01221-21 | SP-GH10-CE | 717 | Hypothetical protein (*Bacteroides cellulosilyticus*,EEF91240) | B | 0.0 | 492/726=67% |
| contig212-01221-23 | SP-GH10-P(-P)-CE | 716 | Hypothetical protein (*Bacteroides eggerthii,EEC53451*) | B | 0.0 | 490/720=68% |
| Contig217-00001-4 | (SP)-GH26 | 366 | Putative β-mannanase (unidentified microorganism,CAJ19138)  Hypothetical protein (*Bacteroides dorei*,EEB26898) | B | 1e-155  9e-83 | 254/354=71%  159/364=43% |
| Contig217-00001-6 | (SP)-GH26 | 396 | β-mannanase (uncultured microorganism,ADB80099)  glycoside hydrolase family protein (*Bacteroides sp.* D2, ZP_05759212) | B | 1e-150  1e-54 | 252/348=72%  144/383=37% |
| Contig217-00011-2 | SP-GH26 | 234 | mannanase-xyloglucanase (uncultured bacterium,ADA62505)  Hypothetical protein (*Bacteroides ovtus*,EDO12203) | B | 1e-43  2e-41 | 97/216=44%  90/199=45% |
| Contig217-00011-41 | SP-GH26 | 417 | Hypothetical protein (*Bacteroides ovtus*,EDO12203) | B | 5e-74 | 155/346=44% |
| contig404-00078-9 | SP-GH26 | 391 | Hypothetical protein (*Bacteroides uniformis*,EDO52278) | B | 1e-147 | 245/392=62% |
| contig310-00038-9 | SP-GH26 | 492 | Hypothetical protein (*Bacteroides cellulosilyticus*,EEF90129) | B | 1e-132 | 218/368=59% |
| contig310-00038-17 | GH26 | 360 | β-mannanase (uncultured microorganism,ADB80101)  Hypothetical protein (*Bacteroides ovatus*,EDO12202) | B | 1e-176  2e-78 | 284/360=78%  153/356=42% |
| contig310-00038-20 | (SP)-GH26 | 471 | Endoglycosidase precursor (uncultured bacterium,ABB46200)  β-1,4-endoglucanase (*Prevotella bryantii*,AAC97596) | B | 1e-122  1e-121 | 215/337=63%  201/344=58% |
| contig212-01208-36 | SP-GH26 | 747 | Putative mannanase (*Prevotella copri*, EFB36843) | B | 1e-128 | 223/422=52% |
| contig404-00010-14 | SP-Acid_phosphat-GH28 | 903 | Hypothetical protein PRU2549(*Prevotella ruminicola*,ADE81266) | B | 1e-150 | 258/423=60% |
| contig212-00030-8 | SP-GH28 | 441 | Galacturan 1,4-α-galcturonidase (*Prevotella buccae*,EFC75217) | B | 1e-132 | 235/438=53% |
| contig212-00030-12 | P-P-GH28 | 1021 | Polygalactuonase (pectinase) (*Bacteroides vulgatus*,EFG17452) | B | 0.0 | 579/1064=54% |
| contig212-00030-15 | SP-GH28 | 471 | Hypothetical protein (*Bacteroides intestinalis*,EDV05149) | B | 1e-157 | 257/444=57% |
| Contig217-00004-38 | GH53(-P) | 350 | Hypothetical protein (*Bacteroides cellulosilyticus*,EEF86974) | B | 1e-137 | 221/348=63% |
| Contig217-00005-26 | SP-P-(P)-GH53 | 380 | Hypothetical protein (*Bacteroides dorei*,EEB25283) | B | 1e-72 | 151/396=38% |
| contig404-00078-23 | SP-GH53 | 346 | Arabinogalactan endo-1,4-β-galactosidase (*Prevotella tannerae*, EEX72059) | B | 4e-79 | 156/317=49% |
| contig210-00110-15 | GH53 | 439 | Glycosyl hydrolase 53 (*Roseburia intestinalis*,EEV00392) | F | 1e-143 | 254/441=59% |
| contig212-00025-41 | SP-Chlorophyllase-GH53 | 676 | beta xylanase (*Bacteroides sp.* D4, EEO47146) | B | 4e-53 | 105/277=37% |
| Contig218-00786-1 | Alpha-L-AF_C | 494 | alpha-N-arabinofuranosidase (*Prevotella ruminicola* 23, ADE81049) | B | 0.0 | 396/494=80% |
| Contig217-00005-12 | **SP-CBM4_9_Alpha-L-AF_C** | 656 | alpha-L-arabinofuranosidase A precursor (uncultured bacterium URE4, ACM91037)  hypothetical protein (*Bacteroides intestinalis* DSM 17393, EDV03641) | B | 0.0  0.0 | 491/638=76%  491/638=76% |
| contig404-00010-27 | SP- Alpha-L-AF_C | 665 | alpha-N-arabinofuranosidase (*Prevotella ruminicola* 23, ADE81803) | B | 0.0 | 484/656=73% |
| Contig217-00011-54 | SP- Alpha-L-AF_C | 818 | hypothetical protein BACCAC_03228 (*Bacteroides caccae* ATCC 43185, edm19479) | B | 0.0 | 422/790=53% |
| contig212-00020-22 | DUF303- Alpha-L-AF_C | 981 | alpha-L-arabinofuranosidase (*Bacteroides eggerthii* 1_2_48FAA, EFV31418) | B | 0.0 | 705/982=71% |
| contig201-00197-1 | **SP-CBM4_9_Alpha-L-AF_C** | 739 | alpha-N-arabinofuranosidase (*Prevotella ruminicola* 23, ADE81175) | B | 1e-174 | 333/767=43% |
| contig404-00003-57 | SP- Alpha-L-AF_C | 527 | alpha-N-arabinofuranosidase (*Prevotella ruminicola* 23, ADE81534) | B | 0.0 | 454/526=86% |
| contig201-00199-5 | SP-DUF303---Alpha-L-AF_C | 843 | family 51 glycosyl hydrolase (*Prevotella ruminicola* 23, ADE81862) | B | 0.0 | 518/836=61% |
| contig308-00761-1 | Alpha-L-AF_C | 241 | alpha-N-arabinofuranosidase (*Prevotella ruminicola* 23, ADE81049) | B | 1e-109 | 184/241=76% |
| contig212-00020-23 | **SP-GH54-NPCBM_assoc** | 810 | xylan 1,4-beta-xylosidase (*Opitutus terrae* PB90-1,ACB75541) | B | 1e-134 | 349/938=37% |
| contig212-00025-29 | SP-GH67m-GH67C | 660 | alpha-glucuronidase (*Prevotella ruminicola* 23, ADE82923) | B | 0.0 | 474/676=70% |
| contig212-01167-37 | SP-GH67M-GH67C | 673 | GH67 (*Bacteroides eggerthii* 1_2_48FAA, EFV29641) | B | 0.0 | 487/661=73% |
| contig201-00004-10 | GH78 | 839 | family 18/alpha-rhamnosidase (*Prevotella ruminicola* 23,ADE83742) | B | 0.0 | 529/845=62% |
| contig201-00196-7 | PfamB812-GH78 | 1060 | hydrolase (*Streptomyces coelicolor* A3(2), CAB53318) | Act | 1e-124 | 336/1055=31% |
| contig210-00006-16 | SP- PfamB812-GH78 | 872 | alpha-L-rhamnosidase (*Chitinophaga pinensis* DSM 2588, ACU61645) | B | 0.0 | 425/911=46% |
| contig210-00033-14 | GH78 | 583 | alfa-L-rhamnosidase (*Prevotella buccae* ATCC 33574, EFU31004) | B | 0.0 | 347/586=59% |
| contig212-00011-2 | SP- PfamB812-GH78 | 961 | hypothetical protein PRABACTJOHN_00690 (*Parabacteroides johnsonii* DSM 18315, EEC97901) | B | 1e-134 | 273/732=37% |
| contig212-00030-5 | SP-GH78 | 1002 | hypothetical protein (*Bacteroides cellulosilyticus* DSM 14838, EEF90138) | B | 0.0 | 499/996=50% |
| contig212-01191-8 | SP-GH78 | 794 | alpha-L-rhamnosidase (*Spirosoma linguale* DSM 74, ADB37990) | B | 0.0 | 381/817=46% |
| contig201-00005-3 | GH1 | 499 | glycosyl hydrolase (*Bacillus pumilus* SAFR-032, ) | F | 1e-140 | 249/500=49% |
| contig404-00075-31 | GH2N-GH2-GH2C | 1236 | GH2 sugar binding (*Paenibacillus sp.* JDR-2, ACT01178) | F | 0.0 | 510/1238=41% |
| contig404-00078-8 | **CBM32-GH2N-GH2-PfamB67** | 1196 | hypothetical protein BACUNI_03890 (*Bacteroides uniformis* ATCC 8492, EDO52277) | B | 0.0 | 641/1229=52% |
| Contig217-00011-3 | SP-GH2N-GH2-GH2C | 932 | beta-galactosidase (*Bacteroides thetaiotaomicron* VPI-5482, AAO79286) | B | 0.0 | 626/937=66% |
| Contig217-00011-26 | SP-GH2N-GH2-GH2C | 776 | family 2 glycosyl hydrolase (*Prevotella ruminicola* 23, ADE81742) | B | 0.0 | 560/771=72% |
| contig212-01160-59 | GH2N-GH2-PfamB67 | 835 | family 2 glycosyl hydrolase (*Prevotella ruminicola* 23, ADE83122) | B | 0.0 | 362/820=44% |
| contig201-00200-11 | GH2N-GH2-GH2C-PfamB9657 | 611 | GH2 sugar binding protein (*Nocardiopsis dassonvillei*, ADH66528) | Act | 0.0 | 382/605=63% |
| contig210-00009-3 | GH2N-GH2-GH2C | 800 | beta-galactosidase (*Subdoligranulum variabile* DSM 15176, EFB76847) | F | 0.0 | 567/798=71% |
| contig210-00108-14 | GH2N-GH2-GH2C | 579 | beta-glucuronidase (*Bryantella formatexigens* DSM 14469, EET58918) | F | 0.0 | 350/596=58% |
| contig310-00017-1 | GH2N-GH2-GH2C-Bgal-small_N | 1182 | glycoside hydrolase family 2 TIM barrel (*Fibrobacter succinogenes*, ACX75381) | Fib | 0.0 | 1030/1165=88% |
| contig310-00426-6 | GH2N-GH2N-GH2-GH2C-Bgal_small_N | 940 | glycoside hydrolase family 2 (*Bacteroides sp.* 4_3_47FAA, EET,18121) | B | 0.0 | 459/741=61% |
| contig310-00426-7 | SP-GH2N-GH2-GH2C-Malectin | 1307 | beta-galactosidase (*Prevotella buccae* D17, EFC76360) | B | 0.0 | 838/1347=62% |
| contig212-00030-18 | SP-GH2N-GH2-GH2C-PfamB18946-PfamB8046 | 1179 | beta-galactosidase (*Bacteroides thetaiotaomicron* VPI-5482, NP_813067) | B | 0.0 | 493/964=51% |
| contig212-01210-15 | **CBM32-GH2N-PfamB7865-GH2-PfamB67(/18592)** | 1125 | sugar binding domain protein (*Prevotella buccalis* ATCC 35310, EFA91088) | B | 0.0 | 587/1116=52% |
| contig404-00010-20 | GH3-GH3C(-PA14) | 894 | family 3 glycosyl hydrolase (*Prevotella ruminicola* 23, ADE83440) | B | 0.0 | 676/849=79% |
| contig404-00027-38 | GH3-GH3C | 772 | Beta-glucosidase (*Bacteroides salanitronis* DSM 18170, YP_004258944) | B | 0.0 | 460/778=59% |
| Contig217-00001-1 | GH3 | 193 | hypothetical protein (*Bacteroides caccae* ATCC 43185, EDM19154) | B | 2e-52 | 98/186=52% |
| contig201-00199-4 | **SP-GH3-GH3C-CBM6-Big_2** | 1262 | GH3 domain protein (*Paludibacter propionicigenes* WB4, YP_004042401) | B | 0.0 | 774/1260=61% |
| contig406-00010-41 | GH3 | 409 | GH3 domain protein (*Marivirga tractuosa* DSM 4126, YP_004055394) | B | 7e-65 | 130/341=38% |
| contig308-00054-5 | GH3C-GH3 | 774 | glycosyl hydrolase (*Sanguibacter keddieii* DSM 10542, ACZ21868) | Act | 0.0 | 418/784=53% |
| contig308-02052-1 | GH3(short) | 119 | beta-hexosaminidase (*Acinetobacter baumannii* AB900, ZP_04660084) | Pro | 2e-64 | 119/119=100% |
| contig310-00120-1 | GH3-GH3C | 399 | beta-glycosidase (*Parabacteroides distasonis* ATCC 8503, ABR44063) | B | 3e-56 | 138/406=33% |
| contig311-00051-1 | GH3-GH3C | 710 | beta-glucosidase (uncultured microorganism, ABU68675)  Beta-glucosidase-related glycosidases (*Alistipes shahii* WAL 8301, CBK63828) | B | 0.0  0.0 | 525/710=73%  480/737=65% |
| contig311-00051-2 | SP-GH3-GH3C | 752 | beta-glucosidase (uncultured microorganism, ADB80109)  GH3 (*Flavobacterium johnsoniae* UW101, ABQ03809) | B | 0.0  1e-170 | 604/752=80%  322/750=42% |
| contig311-00051-14 | SP-GH3-GH3C | 783 | hypothetical protein (*Bacteroides uniformis* ATCC 8492, EDO55247) | B | 0.0 | 421/783=53% |
| contig311-00066-2 | GH3-GH3C | 760 | hypothetical protein (*Bacteroides uniformis* ATCC 8492, EDO52404) | B | 0.0 | 431/773=55% |
| contig311-00229-1 | GH3 | 227 | putative xylosidase (*Prevotella copri* DSM 18205, EFB35044) | B | 1e-116 | 196/226=86% |
| contig311-00405-26 | GH3-GH3C(-PA14) | 868 | family 3 glycosyl hydrolase (*Prevotella ruminicola* 23, ADE83440) | B | 0.0 | 524/874=59% |
| contig311-00405-40 | **SP-GH3-GH3C-CBM6-Big_2** |  | glucan 1,4-beta-glucosidase (*Xanthomonas campestris*, ZP_06489580) | Pro | 1e-116 | 311/889=34% |
| contig311-00410-14 | SP-GH3-GH3C-Beta-lactamase | 962 | GH3 protein (*Dyadobacter fermentans* DSM 18053, ACT95409) | B | 0.0 | 368/951=38% |
| contig212-00020-18 | SP-GH3-GH3C(-PA14) | 868 | GH 3 glycosyl hydrolase (*Prevotella ruminicola* 23, ADE83440) | B | 0.0 | 470/881=53% |
| contig212-00030-28 | GH3-GH3C | 762 | beta-glucosidase (uncultured rumen bacterium, ADD17009)  hypothetical protein BACUNI_00919 (*Bacteroides uniformis* ATCC 8492, EDO055247) | B | 0.0  0.0 | 670/752=89%  504/766=65% |
| contig212-01184-18 | SP-GH3-GH3C | 758 | beta-xylosidase (uncultured rumen bacterium, CAP07659)  GH3 C-terminal domain protein (*Prevotella buccalis* ATCC 35310, EFA93079) | B | 0.0  0.0 | 535/738=72%  363/769=47% |
| contig212-01191-11 | SP-GH3-GH3C | 769 | beta-glucosidase (uncultured rumen bacterium, ADD17009)  hypothetical protein (*Bacteroides uniformis* ATCC 8492, EDO055247) | B | 0.0.  0.0 | 625/770=81%  490/774=63% |
| contig212-01191-12 | SP-GH3-GH3C | 767 | hypothetical protein (*Bacteroides uniformis* ATCC 8492, EDO55247) | B | 0.0 | 448/772=58% |
| contig212-01191-14 | SP-GH3-GH3C | 697 | beta-xylosidase (uncultured rumen bacterium, CAP07659)  GH3 C-terminal domain protein (*Prevotella buccalis* ATCC 35310, EFA93079) | B | 0.0  0.0 | 447/737=60%  347/705=49% |
| contig212-01199-34 | SP-GH3-GH3C | 778 | beta-glucosidase (uncultured rumen bacterium, ADD17009)  hypothetical protein (*Bacteroides uniformis* ATCC 8492, EDO55247) | B | 0.0  0.0 | 613/749=81%  508/783=64% |
| contig212-01199-35 | GH3-GH3C | 767 | hypothetical protein (*Bacteroides uniformis* ATCC 8492, EDO55247) | B | 0.0 | 439/769=57% |
| contig212-01208-27 | GH3-GH3C | 758 | hypothetical protein (*Bacteroides coprocola* DSM 17136, EDV01837) | B | 0.0 | 438/765=57% |
| contig311-00382-1 | GH29 | 111 | alpha-1,3/4-fucosidase family protein (*Prevotella bivia* JCVIHMP010, EFB92937) | B | 4e-12 | 33/63=52% |
| contig212-01224-5 | **SP-GH38-Alpha_mann_mid-GH38C-CBM32** | 1198 | hypothetical protein BACINT_01207 (*Bacteroides intestinalis* DSM 17393, EDV06122) | B | 0.0 | 611/1214=50% |
| contig201-00196-22 | SP-GH39-Beta-lactamase | 905 | hypothetical protein ObacDRAFT_5285 (*Diplosphaera colitermitum* TAV2, EEG18037) | Ver | 2e-73 | 177/468=37=% |
| Contig217-00004-15 | SP-GH42-PfamB5537 | 571 | conserved hypothetical protein (*Prevotella bergensis* DSM 17361, EFA43877) | B | 0.0 | 316/551=57% |
| contig201-00196-8 | SP-GH42 | 498 | hypothetical protein ObacDRAFT_9669 (*Opitutaceae bacterium* TAV2, EEG22510) | Ver | 3e-57 | 165/493=33% |
| contig404-00021-2 | GH42-SLH-SLH-SLH | 779 | Glycoside hydrolase family 42 domain protein (*Victivallis vadensis* ATCC BAA-548, EFB01688) | Ver | 1e-27 | 118/440=26% |
| contig212-00030-10 | SP-GH42-PfamB5537 | 564 | hypothetical protein Oter_1339 (*Opitutus terrae* PB90-1, ACB74624) | Ver | 1e-109 | 224/560=40% |
| contig404-00003-21 | GH43-GH43 | 669 | hypothetical protein PRU_0868 (*Prevotella ruminicola* 23, ADE83202) | B | 1e-162 | 272/330=82% |
| contig404-00075-6 | GH43 | 541 | GH43 (*Clostridium papyrosolvens* DSM 2782, EGD46854) | F | 1e-124 | 238/528=45% |
| contig404-00010-24 | **CE-GH43-CBM6** | 881 | GH43/CBM6 protein (*Prevotella ruminicola* 23, ADE82580) | B | 0.0 | 340/461=73% |
| contig404-00010-21 | GH95-GH43 | 1322 | hypothetical protein PRU_2729 (*Prevotella ruminicola* 23, ADE82580) | B | 0.0 | 516/793=65% |
| contig404-00010-17 | SP-GH43 | 478 | GH 43 glycosyl hydrolase (*Prevotella ruminicola* 23, ADE82026) | B | 0.0 | 347/463=74% |
| contig404-00010-18 | SP-GH43 | 676 | GH 43 glycosyl hydrolase (*Prevotella ruminicola* 23, ADE82665) | B | 0.0 | 378/727=51% |
| Contig213-00065-3 | GH43-GH43 | 322 | xylan 1,4-beta-xylosidase/alpha-N-arabinofuranosidase (*Prevotella ruminicola* 23, ADE81401) | B | 1e-160 | 266/321=82% |
| contig201-00013-15 | GH43-PfamB2334 | 344 | hypothetical protein HMPREF9446_00206 (*Bacteroides fluxus* YIT 12057, EGF59659) | B | 1e-120 | 210/339=61% |
| contig201-00013-18 | GH43 | 807 | hypothetical protein BACINT_01125 (*Bacteroides intestinalis* DSM 17393, EDV06040) | B | 1e-179 | 362/813=44% |
| contig201-00015-45 | SP-GH43 | 189 | family 43 glycosyl hydrolase (*Prevotella ruminicola* 23, ADE83676) | B | 2e-186 | 149/186=80% |
| contig201-00192-4 | **SP-GH43-CBM6** | 778 | Xylan 1,4-beta-xylosidase (*Fibrobacter succinogenes*, ACX75362) | Fib | 0.0 | 738/778=94% |
| contig201-00192-9 | **GH43-CBM6-CBM6** | 722 | Carbohydrate binding family 6 (*Fibrobacter succinogenes*,ACX75357) | Fib | 0.0 | 702/722=97% |
| contig201-00192-10 | **SP-GH43-CBM6-CBM6** | 741 | Carbohydrate binding family 6 (*Fibrobacter succinogenes*,ACX75356) | Fib | 0.0 | 704/741=95% |
| contig201-00192-11 | **SP-GH43-CBM6-CBM6** | 744 | endo-1,4-beta-xylanase (Fibrobacter succinogenes, ADL25514) | Fib | 0.0 | 710/741=95% |
| contig201-00199-7 | **GH43-CBM6** | 727 | hypothetical protein BACCELL_02141 (*Bacteroides cellulosilyticus* DSM 14838, EEF90223) | B | 1e-128 | 220/429=51% |
| contig201-00201-30 | SP-GH43 | 351 | carbohydrate-binding family 6 protein (*Clostridium thermocellum* ATCC 27405, YP_001038591) | F | 1e-107 | 188/296=63% |
| contig210-00009-6 | GH43 | 484 | xylosidase/arabinosidase (*Subdoligranulum variabile* DSM 15176, EFB76844) | F | 0.0 | 347/487=71% |
| contig210-00018-6 | CE-CE-GH43 | 970 | xylosidase/arabinofuranosidase and esterase Xsa43H (*Butyrivibrio proteoclasticus* B316, ADL33049) | F | 0.0, | 960/970=98% |
| contig210-00018-7 | **SP-GH43-CBM6** | 536 | xylosidase/arabinofuranosidase Xsa43A (*Butyrivibrio proteoclasticus* B316, ADL33050) | F | 0.0 | 528/536=98% |
| contig210-00108-7 | GH43 | 511 | Beta-xylosidase (*Eubacterium rectale* DSM 17629, CBK91538) | F | 1e-144 | 262/491=53% |
| contig310-00099-2 | GH43 | 332 | hypothetical protein BACINT_04203 (*Bacteroides intestinalis* DSM 17393, EDV05060) | B | 2e-92 | 175/328=53% |
| contig310-00411-1 | GH43 | 358 | glycoside hydrolase family 43 (*Fibrobacter succinogenes*, ACX75355) | Fib | 1e-151 | 256/351=72% |
| contig310-00424-15 | SP-GH43 | 351 | family 43 glycosyl hydrolase (*Prevotella ruminicola* 23, ADE83310) | B | 1e-130 | 218/351=62% |
| contig311-00409-20 | SP-CE-GH43 | 732 | CHU large protein candidate xylanase/esterase (*Prevotella copri* DSM 18205, EFB34045) | B | 1e-125 | 224/428=52% |
| contig212-00007-19 | SP-GH43 | 569 | glycoside hydrolase family 43 (*Clostridium cellulolyticum* H10, ACL75613) | F | 1e-116 | 228/537=42% |
| contig212-00025-33 | GH43-GH43 | 326 | xylan 1,4-beta-xylosidase/alpha-N-arabinofuranosidase (*Prevotella ruminicola* 23, ADE81401) | B | 1e-153 | 257/325=79% |
| contig212-01167-27 | GH43 | 332 | hypothetical protein BACINT_04203 (*Bacteroides intestinalis* DSM 17393 | B | 1e-130 | 223/328% |
| Contig213-00012-26 | GH43 | 162 | glycoside hydrolase family 43 (*Clostridium cellulolyticum* H10, ACL77726) | F | 5e-30 | 58/95=61% |
| **CBMs** |  |  |  |  |  |  |
| contig201-00192-3 | PfamB3513-Abhydrolase_2-CBM4_9 | 495 | Carbohydrate-binding CenC domain protein (*Fibrobacter succinogenes*, ACX75364) | Fib | 0.0 | 465/498=93% |
| contig201-00204-14 | CBM4_9 | 631 | Alpha-N-arabinofuranosidase (*Opitutus terrae* PB90-1, ACB77608) | Ver | 4e-45 | 159/583=27% |
| contig404-00007-19 | GH16-CBM4_9 | 493 | GH16 (*Paenibacillus sp.* JDR-2, ACS99503) | F | 1e-123 | 230/461=49% |
| contig404-00022-2 | CBM4_9 | 786 | Unknown |  | >1 |  |
| contig310-00212-1 | CBM4_9 | 97 | TonB-dependent receptor (*Teredinibacter turnerae* T7901,ACR14170) | Pro | 0.046 | 31/91=34% |
| contig310-00427-3 | CBM4_9 | 407 | Carbohydrate-binding CenC domain protein (*Fibrobacter succinogenes*, ACX75521) | Fib | 1e-176 | 301/406=74% |
| contig201-00192-2 | PfamB593-Melibiase-CBM6 | 631 | Alpha-galactosidase (*Fibrobacter succinogenes*, ACX75366) | Fib | 0.0 | 589/629=93% |
| contig201-00192-6 | DUF303-CBM6 | 551 | Carbohydrate binding family 6 (*Fibrobacter succinogenes,* ACX75360) | Fib | 0.0 | 495/551=89% |
| contig201-00192-7 | DUF303-CBM6 | 540 | Carbohydrate binding family 6 (*Fibrobacter succinogenes*, AAG36766) | Fib | 0.0 | 494/540=91% |
| contig201-00192-8 | GH30-CBM6 | 691 | Glucuronoarabinoxylan endo-1,4-beta-xylanase (*Fibrobacter succinogenes,* ACX75368) | Fib | 0.0 | 634/691=91% |
| contig210-00111-35 | DUF303-CBM6 | 526 | Carbohydrate binding family 6 (*Fibrobacter succinogenes*, ACX76120) | Fib | 0.0 | 359/536=66% |
| contig310-00001-19 | DUF303-CBM6 | 515 | Carbohydrate binding family 6 (*Fibrobacter succinogenes*, ACX76120) | Fib | 0.0 | 359/508=70% |
| contig310-00001-20 | Pec_lyase-CBM6 | 562 | Pectate lyase/Amb allergen (*Fibrobacter succinogenes*, ACX76150) | Fib | 0.0 | 510/563=90% |
| contig310-00047-1 | CBM6 | 629 | Alpha-L-fucosidase (*Fibrobacter succinogenes*, ACX75382) | Fib | 0.0 | 587/629=93% |
| contig212-00025-45 | GH98M-GH98C-CBM6 | 1213 | hypothetical protein (*Bacteroides ovatus* ATCC 8483, EDO10800) | B | 1e-147 | 280/743=37% |
| contig311-00381-1 | CBM13 | 112 | hypothetical protein (*Ruminococcus torques* ATCC 27756, EDK25059) | F | 4e-08 | 38/111=34% |
| Contig217-00004-31 | PfamB8081-CBM20-GH77 | 826 | putative 4-alpha-glucanotransferase (*Prevotella ruminicola* 23, ADE83753) | B | 0.0 | 574/860=66% |
| Contig217-00018-2 | PfamB8081-CBM20-GH77 | 917 | 4-alpha-glucanotransferase (*Prevotella veroralis* F0319, EEX18691) | B | 0.0 | 579/899=64% |
| contig311-00050-77 | CBM20-Esterase | 599 | putative esterase (*Maricaulis maris* MCS10, ABI67027) | Pro | 2e-23 | 97/305=31% |
| Contig217-00018-1 | F5_F8_Type_C | 514 | F5/8 type C domain protein (*Prevotella timonensis* CRIS 5C-B1, EFA97297) | B | 4e-98 | 204/466=43% |
| contig201-00013-29 | F5_F8_Type_C | 820 | hypothetical protein (*Prevotella ruminicola* 23, ADE82616) | B | 0.0 | 552/812=67% |
| contig212-01191-1 | PfamB239-CBM32-PfamB239 | 562 | GH2 sugar binding (*Dyadobacter fermentans* DSM 18053, ACT92766) | B | 1e-131 | 266/585=45% |
| contig210-00028-17 | CBM34-GH13 | 589 | hypothetical protein (*Ruminococcus gnavus* ATCC 29149, EDN78366) | F | 0.0 | 343/591=58% |
| contig201-00199-2 | PfamB4039-CBM48-PfamB5512-Esterase | 375 | hypothetical protein (*Bacteroides cellulosilyticus* DSM 14838, EEF90227) | B | 1e-100 | 186/367=50% |
| contig404-00075-41 | CBM48-GH13-PfamB375-PfamB2479 | 709 | glycogen debranching enzyme GlgX (*Treponema vincentii* ATCC 35580, EEV20166) | S | 0.0 | 370/725=51% |
| contig310-00038-2 | PfamB4039-CBM48-Esterase | 361 | putative esterase (Zunongwangia profunda SM-A87, ADF53787) | B | 1e-102 | 193/364=53% |
| contig311-00025-13 | CBM48-GH13-Alpha-amylase_C | 655 | 1,4-alpha-glucan branching enzyme (uncultured bacterium URE4, ACM90989)  1,4-alpha-glucan branching enzyme (*Capnocytophaga sputigena* Capno, EEB65317) | B | 0.0  0.0 | 463/664=69%  375/650=57% |
| contig311-00051-4 | PfamB5512-CBM48-Esterase | 391 | probable esterase (Chryseobacterium gleum ATCC 35910, EEI44911) | B | 2e-81 | 175/403=43% |
| contig212-00030-29 | PfamB4039-PfamB5512-CBM48-Esterase | 378 | putative glycosylhydrolase (*Parabacteroides distasonis* ATCC 8503, YP_001304028) | B | 1e-115 | 216/402=53% |
| Contig217-00009-20 | PfamB16589-PeptidaseM23-CBM50 | 323 | peptidase, M23 family (*Prevotella timonensis* CRIS 5C-B1, EFA97598) | B | 1e-82 | 162/326=49% |
| contig308-00201-7 | CBM50 | 173 | hypothetical protein (*Acinetobacter baumannii* AB900, ZP_04662050) | Pro | 1e-95 | 170/173=98% |
| contig308-02314-1 | CBM50 | 54 | soluble lytic murein transglycosylase (*Acinetobacter baumannii* ACICU,ACC56407) | Pro | 1e-22 | 54/54=100% |
| contig310-00059-2 | SLT-CBM50-CBM50 | 563 | hypothetical protein (*Parabacteroides johnsonii* DSM 18315, EEC98414) | B | 1e-109 | 213/459=46% |
| contig311-00042-45 | SLT-CBM50-CBM50 | 572 | hypothetical protein PRABACTJOHN_00167 (*Parabacteroides johnsonii* DSM 18315, EEC98414) | B | 1e-112 | 214/472=45% |
| contig212-00017-46 | SLT-CBM50-CBM50 | 558 | glycoside hydrolase family protein (*Parabacteroides distasonis* ATCC 8503, YP_001303897) | B | 1e-107 | 210/501=41% |
| contig212-01184-20 | Peptidase_M23-CBM50 | 347 | hypothetical protein ALIPUT_00376 (*Alistipes putredinis* DSM 17216, EDS04505) | B | 2e-71 | 138/293=47% |
| contig212-01208-72 | NLPC_P60-CBM50 | 357 | chitinase 3 (*Prevotella tannerae* ATCC 51259, EEX72533) | B | 3e-20 | 63/178=35% |

1, Symbols: Act, Actinobacteria; B, Bacteroidetes; F, Firmicutes; Fib., Fibrobacteres; Pro, Proteobacteria; S, Spirochaetes;Ver, Verrucomicrobia;

Bold faces are the GH proteins that target plant cell wall and carry a CBM.
