# Supplementary material for: Metagenomic Insights into the Fibrolytic Microbiome in Yak Rumen
Source: PLoS One. 2012 Jul 13;7(7):e40430. doi: 10.1371/journal.pone.0040430 (PMC3396655; doi:10.1371/journal.pone.0040430)
Supplement: Table S3 — GH and CBM profiles determined in the BAC library and metagenome of yak rumen microbiome. (DOC) [file pone.0040430.s006.doc]

**Table S3. GH and CBM profiles determined in the** BAC library and metagenome of yak rumen microbiome

| CAZy family | Pfam HMM Name | Pfam Accession | Gene Count | |
| --- | --- | --- | --- | --- |
|  |  |  | BAC | metagenome |
| GHs | | | | |
| GH1 | Glyco_hydro_1 | PF00232 | 1 | 331 |
| GH2 | Glyco_hydro_2 | PF00703 | 13 | 942 |
| GH3 | Glyco_hydro_3 | PF00933 | 24 | 5448 |
| GH4 | Glyco_hydro_4 | PF02056 | 0 | 118 |
| GH5 | Cellulase | PF00150 | 12 | 1302 |
| GH6 | Glyco_hydro_6 | PF01341 | 0 | 0 |
| GH7 | Glyco_hydro_7 | PF00840 | 0 | 0 |
| GH8 | Glyco_hydro_8 | PF01270 | 6 | 174 |
| GH9 | Glyco_hydro_9 | PF00759 | 7 | 767 |
| GH10 | Glyco_hydro_10 | PF00331 | 14 | 2664 |
| GH11 | Glyco_hydro_11 | PF00457 | 0 | 244 |
| GH12 | Glyco_hydro_12 | PF01670 | 0 | 0 |
| GH13 | Alpha-amylase | PF00128 | 27 | 2949 |
| GH14 | Glyco_hydro_14 | PF01373 | 0 | 1 |
| GH15 | Glyco_hydro_15 | PF00723 | 0 | 0 |
| GH16 | Glyco_hydro_16 | PF00722 | 2 | 563 |
| GH17 | Glyco_hydro_17 | PF00332 | 0 | 0 |
| GH18 | Glyco_hydro_18 | PF00704 | 2 | 273 |
| GH19 | Glyco_hydro_19 | PF00182 | 0 | 0 |
| GH20 | Glyco_hydro_20 | PF00728 | 2 | 1107 |
| GH22 | Lys | PF00062 | 0 | 0 |
| GH23 | SLT | PF01464 | 7 | 562 |
| GH24/104 | Phage_lysozyme | PF00959 | 2 | 28 |
| GH25 | Glyco_hydro_25 | PF01183 | 0 | 459 |
| GH26 | Glyco_hydro_26 | PF02156 | 9 | 537 |
| GH27 | Melibise | PF02065 | 5 | 1494 |
| GH28 | Glyco_hydro_28 | PF00295 | 4 | 244 |
| GH29 | Alpha_L_fucos | PF01120 | 1 | 899 |
| GH30 | Glyco_hydro_30 | PF02055 | 3 | 113 |
| GH31 | Glyco_hydro_31 | PF01055 | 8 | 3638 |
| GH32 | Glyco_hydro_32N | PF00251 | 1 | 880 |
| GH33 | BNR | PF02012 | 3 | 30 |
| GH34 | Neur | PF00064 | 0 | 0 |
| GH35 | Glyco_hydro_35 | PF01301 | 0 | 468 |
| GH36 | BLAST SEARCH |  | 0 | 0 |
| GH37 | Trehalase | PF01204 | 3 | 22 |
| GH38 | Glyco_hydro_38 | PF01074 | 1 | 90 |
| GH39 | Glyco_hydro_39 | PF01229 | 1 | 159 |
| GH42 | Glyco_hydro_42 | PF02449 | 4 | 207 |
| GH43 | Glyco_hydro_43 | PF04616 | 28 | 2313 |
| GH44 | BLAST SEARCH |  | 0 | 0 |
| GH45 | Glyco_hydro_45 | PF02015 | 1 | 13 |
| GH46 | Glyco_hydro_46 | PF01374 | 0 | 0 |
| GH47 | Glyco_hydro_47 | PF01532 | 0 | 0 |
| GH48 | Glyco_hydro_48 | PF02011 | 0 | 32 |
| GH49 | Glyco_hydro_49 | PF03718 | 0 | 0 |
| GH50 | BLAST SEARCH |  | 0 | 0 |
| GH51 | BLAST SEARCH |  | 9 | - |
| GH52 | Glyco_hydro_52 | PF03512 | 0 | 0 |
| GH53 | Glyco_hydro_53 | PF07745 | 5 | 1066 |
| GH54 | ArabFuran-catal | PF09206 | 1 | 111 |
| GH55 | BLAST SEARCH |  | 0 | - |
| GH56 | Glyco_hydro_56 | PF01630 | 0 | 0 |
| GH57 | Glyco_hydro_57 | PF03065 | 3 | 250 |
| GH58 | BLAST SEARCH |  | 0 | - |
| GH59 | Glyco_hydro_59 | PF02057 | 2 | 4 |
| GH61 | Glyco_hydro_61 | PF03443 | 0 | 0 |
| GH62 | Glyco_hydro_62 | PF03664 | 0 | 0 |
| GH63 | Glyco_hydro_63 | PF03200 | 0 | 17 |
| GH64 | BLAST SEARCH |  | 0 | - |
| GH65 | Glyco_hydro_65m | PF03632 | 0 | 32 |
| GH66 | BLAST SEARCH |  | 0 | - |
| GH67 | Glyco_hydro_67M | PF07488 | 2 | 1090 |
| GH68 | Glyco_hydro_68 | PF02435 | 0 | 0 |
| GH70 | Glyco_hydro_70 | PF02324 | 0 | 4 |
| GH71 | Glyco_hydro_71 | PF03659 | 0 | 0 |
| GH72 | Glyco_hydro_72 | PF03198 | 0 | 0 |
| GH73 | Glucosaminidse | PF01832 | 0 | 0 |
| GH74 | BLAST SEARCH |  | 2 | - |
| GH75 | Chitosanase | PF07335 | 0 | 0 |
| GH76 | Glyco_hydro_76 | PF03663 | 2 | 39 |
| GH77 | Glyco_hydro_77 | PF02446 | 6 | 1739 |
| GH78 | Bac_rhamnosid | PF05592 | 7 | 426 |
| GH79 | Glyco_hydro_79n | PF03662 | 0 | 0 |
| GH80 | BLAST SEARCH |  | 0 | - |
| GH81 | Glyco_hydro_81 | PF03639 | 0 | 1 |
| GH82 | BLAST SEARCH |  | 0 | - |
| GH83 | HN | PF00423 | 0 | 0 |
| GH84 | Hyaluronidase_2 | PF07555 | 0 | 0 |
| GH85 | Glyco_hydro_85 | PF03644 | 0 | 1 |
| GH86 | BLAST SEARCH |  | 0 | - |
| GH87 | BLAST SEARCH |  | 0 | - |
| GH88/105 | Glyco_hydro_88 | PF07470 | 4 | 675 |
| GH89 | NAGLU | PF05089 | 5 | 320 |
| GH90 | BLAST SEARCH |  | 0 | - |
| GH91 | BLAST SEARCH |  | 0 | - |
| GH92 | Glyco_hydro_92 | PF07971 | 2 | 2378 |
| GH93 | BLAST SEARCH |  | 0 | - |
| GH94 | BLAST SEARCH |  | 2 | - |
| GH95 | BLAST SEARCH |  | 2 | - |
| GH96 | BLAST SEARCH |  | 0 | - |
| GH97 | BLAST SEARCH | PF10566 | 12 | - |
| GH98 | Glyco_hydro_98M | PF08306 | 1 | 95 |
| GH99 | BLAST SEARCH |  | 0 | - |
| GH100 | Glyco_hydro_100 | PF12899 | 0 | 0 |
| GH101 | Glyco_hydro_101 | PF12905 | 0 | 224 |
| GH102 | MltA | PF03562 | 0 | 1 |
| GH103 | TIGR:MltB | TIGR02282 | 0 | 0 |
| GH106 | BLAST SEARCH |  | 4 | - |
| GH107 | BLAST SEARCH |  | 0 | - |
| GH108 | DUF847 | PF05838 | 1 | 19 |
| GH109 | BLAST SEARCH |  | 0 | - |
| GH110 | BLAST SEARCH |  | 0 | - |
| Total | | | 263 | 37563 |
| CBMs | | | | |
| CBM1 | CBM_1 | PF00734 | 0 | 0 |
| CBM2 | CBM_2 | PF00553 | 0 | 76 |
| CBM3 | CBM_3 | PF00942 | 0 | 47 |
| CBM4/CBM9 | CBM_4_9 | PF02018 | 9 | 115 |
| CBM5/CBM12 | CBM_5_12 | PF02839 | 0 | 6 |
| CBM6 | CBM_6 | PF03422 | 18 | 294 |
| CBM10 | CBM_10 | PF02013 | 0 | 0 |
| CBM11 | CBM_11 | PF03425 | 0 | 1 |
| CBM13 | Ricin_B_lectin | PF00652 | 1 | 25 |
| CBM14 | CBM_14 | PF01607 | 0 | 0 |
| CBM15 | CBM_15 | PF03426 | 0 | 1 |
| CBM17/CBM28 | CBM_17_28 | PF03424 | 0 | 0 |
| CBM18 | Chitin_bind_1 | PF00187 | 0 | 0 |
| CBM19 | CBM_19 | PF03427 | 0 | 0 |
| CBM20 | CBM_20 | PF00686 | 3 | 92 |
| CBM21 | CBM_21 | PF03370 | 0 | 1 |
| CBM25 | CBM_25 | PF03423 | 0 | 2 |
| CBM27 | CBM27 | PF09212 | 0 | 0 |
| CBM32/CBM47 | F5_F8_type_C | PF00754 | 6 | 150 |
| CBM33 | Chitin_bind_3 | PF03067 | 0 | 0 |
| CBM34 | Alpha_amylase_N | PF02903 | 1 | 16 |
| CBM40 | Sialidase | PF02973 | 0 | 0 |
| CBM41 | PUD | PF03714 | 0 | 2 |
| CBM42 | AbfB | PF05270 | 0 | 0 |
| CBM43 | X8 | PF07983 | 0 | 0 |
| CBM48 | CBM_48 | PF02922 | 6 | 458 |
| CBM49 | CBM_49 | PF09478 | 0 | 0 |
| CBM50 | LysM | PF01476 | 8 | 663 |
| CBM51 | MPCBM | PF08305 | 0 | 0 |
| Total | | | 52 | 1949 |
