# Supplementary material for: Metagenomic Insights into the Fibrolytic Microbiome in Yak Rumen
Source: PLoS One. 2012 Jul 13;7(7):e40430. doi: 10.1371/journal.pone.0040430 (PMC3396655; doi:10.1371/journal.pone.0040430)
Supplement: Table S4 — Identified enzymatic activities of the proteins from various glycoside hydrolase families those targeting plant-cell wall in Carbohydrate-Active enZYmes Database (CAZy). (DOC) [file pone.0040430.s007.doc]

**Table S4**. Identified enzymatic activities of the proteins from various glycoside hydrolase families those targeting plant-cell wall in Carbohydrate-Active enZYmes Database (CAZy)

| GH family | Identified activities (the Enzyme Commission number)^§^ |
| --- | --- |
| Cellulase | |
| GH5 | chitosanase (EC 3.2.1.132); β-mannosidase (EC 3.2.1.25); **cellulase (EC 3.2.1.4)**; glucan β-1,3-glucosidase (EC 3.2.1.58); licheninase (EC 3.2.1.73); glucan endo-1,6-β-glucosidase (EC 3.2.1.75); mannan endo-β-1,4-mannosidase (EC 3.2.1.78); endo-β-1,4-xylanase (EC 3.2.1.8); cellulose β-1,4-cellobiosidase (EC 3.2.1.91)*; β-1,3-mannanase (EC 3.2.1.-); xyloglucan-specific endo-β-1,4-glucanase (EC 3.2.1.151); mannan transglycosylase (EC 2.4.1.-); endo-β-1,6-galactanase (EC 3.2.1.164); endoglycoceramidase (EC 3.2.1.123); β-primeverosidase (EC 3.2.1.149) |
| GH6 | **endoglucanase (EC 3.2.1.4)**; cellobiohydrolase (EC 3.2.1.91) |
| GH7 | endo-β-1,4-glucanase (EC 3.2.1.4); **reducing end-acting cellobiohydrolase (EC 3.2.1.176)**; chitosanase (EC 3.2.1.132); endo-β-1,3-1,4-glucanase (EC 3.2.1.73) |
| GH9 | **endoglucanase (EC 3.2.1.4)**; cellobiohydrolase (EC 3.2.1.91)^#^; β-glucosidase (EC 3.2.1.21); exo-β-glucosaminidase (EC 3.2.1.165) |
| GH44 | **endoglucanase (EC 3.2.1.4)**; xyloglucanase (EC 3.2.1.151) |
| GH45 | endoglucanase (EC 3.2.1.4) |
| GH48 | **reducing end-acting cellobiohydrolase (EC 3.2.1.176)**; endo-β-1,4-glucanase (EC 3.2.1.4); chitinase (EC 3.2.1.14) |
| Endohemicellulases | |
| GH8 | **chitosanase (EC 3.2.1.132);** cellulase (EC 3.2.1.4); licheninase (EC 3.2.1.73); endo-1,4-β-xylanase (EC 3.2.1.8); reducing-end-xylose releasing exo-oligoxylanase (EC 3.2.1.156) |
| GH10 | **endo-1,4-β-xylanase (EC 3.2.1.8)**; endo-1,3-β-xylanase (EC 3.2.1.32) |
| GH11 | xylanase (EC 3.2.1.8) |
| GH12 | **endoglucanase (EC 3.2.1.4)**; xyloglucan hydrolase (EC 3.2.1.151); β-1,3-1,4-glucanase (EC 3.2.1.73); xyloglucan endotransglycosylase (EC 2.4.1.207) |
| GH26 | **β-mannanase (EC 3.2.1.78)**; β-1,3-xylanase (EC 3.2.1.32) |
| GH28 | **polygalacturonase (EC 3.2.1.15)**; exo-polygalacturonase (EC 3.2.1.67); exo-polygalacturonosidase (EC 3.2.1.82); rhamnogalacturonase (EC 3.2.1.171); endo-xylogalacturonan hydrolase (EC 3.2.1.-); rhamnogalacturonan a-L-rhamnopyranohydrolase (EC 3.2.1.40) |
| GH53 | endo-β-1,4-galactanase (EC 3.2.1.89). |
| Debranching enzymes | |
| GH51 | **a-L-arabinofuranosidase (EC 3.2.1.55)**; endoglucanase (EC 3.2.1.4) |
| GH54 | a-L-arabinofuranosidase (EC 3.2.1.55); β-xylosidase (EC 3.2.1.37). |
| GH62 | a-L-arabinofuranosidase (EC 3.2.1.55) |
| GH67 | **a-glucuronidase (EC 3.2.1.139)**; xylan a-1,2-glucuronidase (EC 3.2.1.131) |
| GH78 | a-L-rhamnosidase (EC 3.2.1.40) |
| Oligosaccharide-degrading enzymes | |
| GH1 | **β-glucosidase (EC 3.2.1.21)**; β-galactosidase (EC 3.2.1.23); β-mannosidase (EC 3.2.1.25); β-glucuronidase (EC 3.2.1.31); β-D-fucosidase (EC 3.2.1.38); phlorizin hydrolase (EC 3.2.1.62); exo-β-1,4-glucanase (EC 3.2.1.74); 6-phospho-β-galactosidase (EC 3.2.1.85); 6-phospho-β-glucosidase (EC 3.2.1.86); strictosidine β-glucosidase (EC 3.2.1.105); lactase (EC 3.2.1.108); amygdalin β-glucosidase (EC 3.2.1.117); prunasin β-glucosidase (EC 3.2.1.118); raucaffricine β-glucosidase (EC 3.2.1.125); thioglucosidase (EC 3.2.1.147); β-primeverosidase (EC 3.2.1.149); isoflavonoid 7-O-β-apiosyl-β-glucosidase (EC 3.2.1.161); hydroxyisourate hydrolase (EC 3.-.-.-); β-glycosidase (EC 3.2.1.-) |
| GH2 | **β-galactosidase (EC 3.2.1.23)**; β-mannosidase (EC 3.2.1.25); β-glucuronidase (EC 3.2.1.31); mannosylglycoprotein endo-β-mannosidase (EC 3.2.1.152); exo-β-glucosaminidase (EC 3.2.1.165) |
| GH3 | **β-glucosidase (EC 3.2.1.21)**; xylan 1,4-β-xylosidase (EC 3.2.1.37); β-N-acetylhexosaminidase (EC 3.2.1.52); glucan 1,3-β-glucosidase (EC 3.2.1.58); glucan 1,4-β-glucosidase (EC 3.2.1.74); exo-1,3-1,4-glucanase (EC 3.2.1.-); a-L-arabinofuranosidase (EC 3.2.1.55) |
| GH29 | **a-L-fucosidase (EC 3.2.1.51)**; a-1,3/1,4-L-fucosidase (EC 3.2.1.111) |
| GH35 | **β-galactosidase (EC 3.2.1.23)**; exo-β-glucosaminidase (EC 3.2.1.165) |
| GH38 | **a-mannosidase (EC 3.2.1.24)**; mannosyl-oligosaccharide a-1,3-1,6-mannosidase (EC 3.2.1.114); mannosyl-oligosaccharide a-1,3-mannosidase (EC 3.2.1.-) |
| GH39 | a-L-iduronidase (EC 3.2.1.76); **β-xylosidase (EC 3.2.1.37)** |
| GH42 | β-galactosidase (EC 3.2.1.23) |
| GH43 | **β-xylosidase (EC 3.2.1.37)**; β-1,3-xylosidase (EC 3.2.1.-); **a-L-arabinofuranosidase (EC 3.2.1.55)**; arabinanase (EC 3.2.1.99); xylanase (EC 3.2.1.8); galactan 1,3-β-galactosidase (EC 3.2.1.145) |
| GH52 | β-xylosidase (EC 3.2.1.37) |

^§^，bold faced refer to the main activities that the GH proteins in the defined families in CAZy;

*, only 2 β-1,4-cellobiosidase (EC 3.2.1.91) in 2023 bacterial GH5 enzymes in CAZy;

^#^, only 3 β-1,4-cellobiosidase (EC 3.2.1.91) in 480 bacterial GH5 enzymes in CAZy.
