# Supplementary material for: Metagenomic Insights into the Fibrolytic Microbiome in Yak Rumen
Source: PLoS One. 2012 Jul 13;7(7):e40430. doi: 10.1371/journal.pone.0040430 (PMC3396655; doi:10.1371/journal.pone.0040430)
Supplement: Table S5 — Cellulase and hemicellulase activities of the over expressed proteins from the novel subfamily 6 of GH5 obtained in the yak rumen. (DOC) [file pone.0040430.s008.doc]

**Table S5. Cellulase and hemicellulase activities of the over expressed proteins from the novel subfamily 6 of GH5** obtained in the yak rumen

| ORF Specific activity (U/mg) | | | | |
| --- | --- | --- | --- | --- |
|  | CMC | Avicel | Birch wood | Mannan |
| Contig404-00027-37 | 0.565±0.19 | 0.055±0.01 | 0.48±0.06 | 1.14±0.08 |
| Contig310-00038-19 | 1.66±0.26 | 0.16±0.02 | 6.04±0.31 | 7.88±0.15 |
