# Supplementary material for: Metagenomic Insights into the Fibrolytic Microbiome in Yak Rumen
Source: PLoS One. 2012 Jul 13;7(7):e40430. doi: 10.1371/journal.pone.0040430 (PMC3396655; doi:10.1371/journal.pone.0040430)
Supplement: Information S1 — Supplementary Methods. (DOC) [file pone.0040430.s009.doc]

Information S1:

**Supplementary Methods**

***Rumen sampling.*** Two Qinghai-Tibetan domesticated yak (each ~500 kg in body weight) fitted with rumen fistula were fed for two weeks with wheat stalk diet, the routine diet for yaks during winter, spring and autumn. The diet is composed with 4.8% crude protein, 73% neutral detergent fiber, 49.4% acid detergent fiber, 8.8% lignin, 0.3% calcium, 0.1% phosphorus and 7.6% trace mineralized salt, Thorough the rumen fistula, a mixture of rumen fluid and undigested fiber was collected from two yaks 1-2 hours after morning feeding. The rumen samples (~ 100 ml each) were centrifuged at 30,000g at 4℃ for 30 min and then mixed and stored at –70°C before use.

***DNA Extraction*.** The cell pellet of subsample was resuspended in SET buffer (1 M NaCl, 0.1 M EDTA, 10 mM Tris, pH 8.0) to a cell density of ~10^8^ cells/ml and embedded in low melting point agarose. The agarose plug containing the cells was immersed first in a lysis buffer (10 mM Tris [pH 8.0], 50 mM NaCl, 0.1 M EDTA, 1% Sarkosyl, 0.2% sodium deoxycholate, 1 mg of lysozyme per ml) for 1 h at 37^o^C and then in a restriction digestion buffer (6 mM Tris-HCl, 100 mM NaCl, 1 mM DTT) with HindIII for 20 min at 37^o^C. The DNA digest in the plug was subjected to pulse field gel electrophoresis CHEF MAPPER system (Bio-Rad), and fragments of 50-200 kb in size were recovered from the gel by electroelution.

***Screening of fibrolytic BAC clones*.** Colonies in 384-well plate were transferred to 96-well plates using a 384-well transfer device (V&P Scientific Inc. San Diego CA) and then to large Petri dishes (150×150mm) containing LB agar with 12.5 mg·mL^-1^ of chloromphenical for screening those containing various fibrolytic enzymes genes. Esterase activity was detected using α-naphthyl acetate as the indicator , xylase, cellulase and lipase activities were screened on the LB agar containing 0.5% (w/v) birch wood xylan, carboxymethyl cellulose (CMC) and glycerol tributyrate (Sigma), respectively. Positive clones were identified by the clearing zone or ‘halo’ upon Congo red staining after incubated for 16 h . The BAC plasmids were purified and retransformed into *E. coli* EPI300 to further confirm the activities.

***Characterization of the cellulases in novel subfamily 6 of GH5*.** The substrate specificities of the purified proteins contig310-00038-19 and contig404-00027-37 were measured on the polysaccharides (1% (w/ v)) as follows, carboxymethyl cellulose (CMC), Avicel (Fluka), locust bean (Fluka), pectin (Fluka), oat spelt xylan (Sigma) and birch wood xylan (Sigma), as well as p-nitrophenyl-β-D-cellobioside (pNPC, 2.5 mmol·L^-1^). For determination of the activity on Avicel and pNPC, 0.l g of the purified protein was used. Enzymatic activity was determined by reducing sugar release from the substrates in 0.5 ml citrate/phosphate buffer, pH 4.0 and pH 4.5 and incubated at 40 ^o^C for 30 min as described by Miller (1959) . One unit (U) of activity was defined as the amount of enzyme releasing one micromole of reducing sugar per minute from the substrate. The concentrations of two recombined enzymes were measured by the method of Bradford (1976) .

**References:**

1. Ferrer M, Chernikova TN, Timmis KN, Golyshin PN (2004) Expression of a temperature-sensitive esterase in a novel chaperone-based *Escherichia coli* strain. Appl Environ Microbiol 70: 4499-4504.

2. Teather RM, Wood PJ (1982) Use of Congo red-polysaccharide interactions in enumeration and characterization of cellulolytic bacteria from the bovine rumen. Appl Environ Microbiol 43: 777-780.

3. Miller GL (1959) Use of dinitrosalicylic acid reagent for determination of reducing sugar. Anal Chem 31: 426-428.

4. Bradford MM (1976) A rapid and sensitive method for the quantitation of microgram quantities of protein utilizing the principle of protein-dye binding. Anal Biochem 72: 248-254.
